# Spatial organization of neutrophil and macrophage populations in surgically treated hepatocellular carcinoma: a practical tissue-based immunophenotyping study

**DOI:** 10.3389/fimmu.2026.1982507

**Published:** 2026-10-07

**Authors:** Konstantinos Arvanitakis, Christos Topalidis, Vasileios Papadopoulos, Eirini Giannousi, Georgios Kalopitas, Antonios Chatzigeorgiou, Georgios Katsanos, Anastasia Maria Natsi, Ioannis Mitroulis, Stella Arelaki, Chrysoula Gouta, Evangelia Katsiki, Prodromos Hytiroglou, Georgios Tsoulfas, Triantafyllia Koletsa, Georgios Germanidis

**Affiliations:** 1 Division of Gastroenterology and Hepatology, First Department of Internal Medicine, AHEPA University Hospital, Aristotle University of Thessaloniki, Thessaloniki, Greece; 2 Department of Pathology, School of Medicine, Aristotle University of Thessaloniki, Thessaloniki, Greece; 3 Laboratory of Anatomy, Medical School, Democritus University of Thrace, Alexandroupolis, Greece; 4 Department of Physiology, Medical School, National and Kapodistrian University of Athens, Athens, Greece; 5 Department of Transplantation Surgery, Hippokration General Hospital, School of Medicine, Aristotle University of Thessaloniki, Thessaloniki, Greece; 6 Laboratory of Molecular Hematology, Democritus University of Thrace, Alexandroupolis, Greece; 7 Department of Pathology, Democritus University of Thrace, Alexandroupolis, Greece; 8 Department of Pathology, Hippokration General Hospital, School of Medicine, Aristotle University of Thessaloniki, Thessaloniki, Greece

**Keywords:** CD177, hepatocellular carcinoma, neutrophil heterogeneity, spatial immunophenotyping, tumor microenvironment, tumor-associated neutrophils

## Abstract

**Background and aims:**

Hepatocellular carcinoma (HCC) has a spatially heterogeneous immune microenvironment, but the compartment-specific distribution of tumor-associated neutrophils (TANs) and its relationship with systemic inflammation remain incompletely defined. This study characterized CD177-defined neutrophil populations and other immune components across HCC tissue compartments and examined their associations with peripheral neutrophil-to-lymphocyte ratio (NLR).

**Methods:**

Paired tumor center (TC), tumor margin/interface (IM), and adjacent non-neoplastic liver (NNL) specimens from 28 patients with HCC were analyzed using immunofluorescence microscopy and quantitative immunohistochemistry. Total neutrophil elastase-positive (NE^+^) and CD177^+^NE^+^ neutrophils, CD8^+^ lymphocytes, CD68^+^ and CD163^+^ macrophages, and CD34^+^ microvascular density were quantified.

**Results:**

Total NE^+^ neutrophil density differed across compartments and was higher in both tumor-associated compartments than in NNL (median: TC, 46.40 cells/mm²; IM, 39.65 cells/mm²; NNL, 8.80 cells/mm²; *P* < 0.001). CD177^+^NE^+^ density showed a modest compartmental effect and was higher in the TC than at the IM (RR 2.68, 95% CI 1.20–6.02; Holm-adjusted *P* = 0.050). In contrast, the CD177^+^ fraction among total NE^+^ neutrophils was markedly lower in both tumor-associated compartments than in NNL (median: TC, 17.63%; IM, 10.90%; NNL, 88.08%; *P* < 0.001)among, accompanied by substantial enrichment of estimated NE^+^CD177^-^ neutrophils. CD8^+^ lymphocytes preferentially accumulated at the IM, whereas CD34^+^ microvascular density followed a TC>IM>NNL gradient. CD68^+^ macrophages were more abundant in NNL, while CD163^+^ macrophage density showed a modest overall compartment effect (mixed-effects *P* = 0.037), and the CD163/CD68 macrophage-polarization index was higher in the TC than at the IM (*P* = 0.009). In exploratory analyses, higher preoperative NLR was associated with the CD163/CD68 macrophage-polarization index specifically in the TC (ρ = 0.451, *P* = 0.016; q = 0.048), but not with neutrophil abundance, CD177-defined distribution, or patient-level neutrophil spatial-distribution indices.

**Conclusions:**

HCC exhibits marked spatial myeloid heterogeneity, characterized by neutrophil enrichment in tumor-associated compartments, compartmental differences in CD177-defined populations, and compartment-specific macrophage polarization. These findings, generated using a practical tissue-based immunophenotyping approach applicable to routinely processed specimens, warrant validation in larger mechanistic studies.

## 1 Introduction

Primary liver cancer is the sixth most frequent malignancy and the third leading cause of cancer-related mortality worldwide, with more than 900,000 new cases and 800,000 deaths annually (1, 2). Hepatocellular carcinoma (HCC), which accounts for approximately 75–90% of primary liver cancers, typically develops in the setting of chronic liver inflammation and cirrhosis and is characterized by a complex immune microenvironment (3). Interactions between malignant hepatocytes, stromal and vascular components, and infiltrating immune cells influence tumor progression, therapeutic response, and clinical outcome (4). Accordingly, increasing attention has shifted from individual immune cell populations toward their spatial organization and functional interactions within the tumor ecosystem (5, 6).

Among these populations, neutrophils have emerged as important regulators of HCC biology. Once regarded primarily as short-lived innate effector cells, neutrophils are now recognized as highly plastic cells capable of adopting diverse phenotypic and functional states in response to local microenvironmental signals (7). Tumor-associated neutrophils (TANs) have been implicated in extracellular matrix remodeling, angiogenesis, immune suppression, invasion, and metastatic progression (8). Single-cell and spatial profiling studies further demonstrate that TANs are not a homogeneous population but comprise dynamic states shaped by the surrounding microenvironment (9, 10). These observations have shifted the focus from neutrophil abundance alone toward understanding their phenotypic heterogeneity and spatial organization within human cancers.

Spatial organization is a fundamental determinant of immune cell behavior within solid tumors, and emerging spatial-omics studies in HCC indicate that the localization and cellular interactions of immune and stromal populations may provide biological information beyond cellular abundance alone (11, 12). Distinct anatomical compartments, including the tumor center (TC), tumor margin/interface (IM), and adjacent non-neoplastic tissue (NNL), differ in oxygen tension, cytokine composition, extracellular matrix architecture, vascularization, and cellular interactions, generating immunological niches that influence immune cell phenotype and function (13, 14). Consequently, analyses that disregard spatial localization may overlook biologically relevant compartment-specific differences. Although spatial heterogeneity of lymphoid and myeloid populations is increasingly recognized in HCC, the spatial organization and phenotypic heterogeneity of tumor-associated neutrophils remain poorly understood. CD177, a glycosylphosphatidylinositol (GPI)-anchored surface glycoprotein expressed by a subset of mature neutrophils, has emerged as a marker of neutrophil heterogeneity. Rather than defining a single functional phenotype, CD177 has been associated with differences in migratory capacity, endothelial interactions, and transcriptional profiles (15, 16), while its expression may vary in response to inflammatory or tumor-associated signals (17). However, the spatial distribution of CD177-defined neutrophil subsets within HCC and their relationship with other components of the tumor microenvironment remain poorly characterized.

It has been demonstrated that a higher proportion of circulating CD177-positive neutrophils was independently associated with improved recurrence-free and overall survival following curative treatment for HCC (18), raising the question of how CD177-defined neutrophil populations are distributed across distinct regions of the tumor microenvironment. Macrophages constitute another major component of the HCC myeloid landscape, with their phenotype shaped by local tumor-derived and inflammatory signals (19). Relative CD68 and CD163 expression may reflect compartment-specific macrophage composition. However, the relationship between local myeloid populations and systemic inflammation remains unclear (20). The peripheral neutrophil-to-lymphocyte ratio (NLR) is an established prognostic inflammatory marker in HCC, but whether it reflects tissue neutrophil accumulation, neutrophil phenotype, macrophage polarization, or broader systemic inflammation is uncertain (21, 22). Examining these relationships across matched tissue compartments may help distinguish systemic inflammation from local immune organization.

This study aimed to characterize the spatial organization of the immune microenvironment across paired tumor center (TC), tumor margin/interface (IM), and adjacent non-neoplastic liver (NNL) in patients with surgically treated HCC. Using conventional immunohistochemistry combined with double immunofluorescence on routinely processed formalin-fixed paraffin-embedded (FFPE) tissue, we applied a practical and reproducible tissue-based approach that does not require high-dimensional omics technologies. Emphasis was placed on defining the compartment-specific distribution of CD177-defined tumor-associated neutrophil populations and determining whether systemic inflammatory status, assessed by peripheral NLR, is differentially associated with neutrophil composition and macrophage polarization within distinct tissue compartments.

## 2 Materials and methods

### 2.1 Study design and patient cohort

This retrospective, multicenter translational study investigated the spatial organization and CD177-defined composition of tumor-associated neutrophils within the HCC immune microenvironment. Consecutive patients undergoing curative-intent liver resection (LR) or orthotopic liver transplantation (LT) for histologically confirmed HCC were identified from institutional databases at two tertiary referral centers in Greece: the First Department of Internal Medicine, AHEPA University Hospital, and the Department of Transplantation Surgery, Hippokration General Hospital, both affiliated with Aristotle University of Thessaloniki. Patients treated between January 2013 and December 2024 were screened for eligibility.

Eligible patients were adults (≥18 years) with HCC who underwent curative surgical treatment and had archived formalin-fixed paraffin-embedded (FFPE) tissue available for spatial immune profiling. HCC was diagnosed histologically or according to the Liver Imaging Reporting and Data System (LI-RADS) criteria when appropriate (23). Tumor stage was assigned according to the Barcelona Clinic Liver Cancer (BCLC) classification (24). Clinicopathological information was retrieved retrospectively from electronic medical records. Recorded variables included demographic characteristics, underlying liver-disease etiology, Child–Pugh class and score, Model for End-Stage Liver Disease (MELD) score, preoperative alpha-fetoprotein (AFP), platelet count, absolute neutrophil and lymphocyte counts, tumor characteristics, treatment modality, prior locoregional treatment, and previous hepatic resection. Prior locoregional treatment included transarterial chemoembolization (TACE), radiofrequency ablation (RFA), and radiotherapy. Preoperative NLR was calculated by dividing the absolute neutrophil count by the absolute lymphocyte count. Surgical treatment was determined through multidisciplinary evaluation of liver function, portal-hypertension status, technical resectability, tumor burden, transplant eligibility, AFP concentration, and previous response to locoregional treatment. Hepatic resection was selected for technically resectable disease in patients with preserved hepatic reserve; all resection patients in this cohort had Child-Pugh class A liver function. Liver transplantation was selected for patients with cirrhosis and tumors within Milan criteria or, in selected cases, beyond Milan criteria following multidisciplinary evaluation and, where applicable, pretransplant locoregional treatment. For liver-transplant recipients, the interval from HCC diagnosis to transplantation was calculated from the recorded diagnosis and procedure dates.

Patients were excluded if they were younger than 18 years of age, underwent non-curative surgery (R2 resection or palliative procedures), had concurrent extrahepatic malignancy, or lacked essential clinicopathological information. Archived FFPE tissue blocks were independently reviewed by two experienced hepatopathologists. Cases with complete tumor necrosis, poor fixation, inadequate tissue preservation or insufficient representative tumor and adjacent liver tissue for spatial analysis were excluded prior to immunophenotyping. Of the 31 initially eligible patients, three were excluded because of non-evaluable tissue specimens, resulting in a final analytical cohort of 28 patients for spatial immunophenotyping.

The study involving human participants was reviewed and approved by the Institutional Review Board and Ethics Committee of Aristotle University of Thessaloniki (approval nos. 3.683/18-1–2022 and 17285/11-4-2023) and was conducted in accordance with the Declaration of Helsinki and applicable institutional requirements (25, 26). Archived formalin-fixed, paraffin-embedded tissue specimens and clinical data obtained during routine clinical care were used retrospectively. The requirement for written informed consent was waived by the responsible ethics committee because of the retrospective design and the anonymized analysis of clinical data and archived tissue.

### 2.2 Definition of tissue compartments

To investigate compartment-dependent immune organization, three anatomically and biologically distinct tissue compartments were evaluated in every patient: the tumor center (TC), the tumor margin/interface (IM), and the adjacent non-neoplastic liver (NNL). These compartments were selected because they represent distinct microenvironmental niches characterized by differences in tumor architecture, vascularization, inflammatory signaling and immune cell interactions. The tumor center was defined as viable intratumoral tissue located away from the IM, whereas the latter represented the transition zone extending 0.5 mm on each side of the tumor-host interface. Adjacent non-neoplastic liver was sampled from uninvolved hepatic tissue remote from the tumor and served as the patient-specific reference. Analyzing paired compartments from the same individual minimized inter-patient variability and enabled direct assessment of spatial immune organization within the HCC microenvironment.

### 2.3 Immunohistochemistry

Unstained 4-μm-thick tissue sections cut from paraffin blocks were mounted on Superfrost plus slides (Thermo Fisher Scientific, Waltham, MA, USA). Immunohistochemistry was performed in an automated immunohistochemistry device (Autostainer Link 48, Agilent/Dako, Santa Clara, CA, USA) using the EnVision FLEX detection system (K8023, Agilent/Dako, Santa Clara, CA, USA) with 3,3’-diaminobenzidine (DAB) as chromogen and Mayer hematoxylin as counterstain. Prior to staining the FFPE tissue sections were subjected to deparaffinization, and hydration followed by heat-induced epitope retrieval (HIER1) in a PT Link (Agilent/Dako, Santa Clara, CA, USA). Endogenous peroxidase was blocked with EnVision FLEX Peroxidase-Blocking Reagent (SM801) included in the kit. Finally, sections were incubated for 20 min with the following primary antibodies: anti-CD163 (GHI/61: sc-20066, mouse monoclonal antibody, Santa Cruz Biotechnology, Santa Cruz, CA, USA, dilution 1:100), anti-CD68 (KP1, mouse monoclonal antibody, Agilent/Dako, Santa Clara, CA, USA, dilution 1:1000), anti-CD8 (C8/144B, mouse monoclonal antibody, Agilent/Dako, Santa Clara, CA, USA, dilution 1:100), and anti-CD34 (QBEnd 10, mouse monoclonal antibody, Agilent/Dako, Santa Clara, CA, USA, dilution 1:100) (**Supplementary Table S1**).

### 2.4 Evaluation of immunohistochemical stains

Evaluation of immunohistochemical stains was conducted using the Nikon Eclipse E600 optical microscope. For each compartment, three representative high-power fields (×20 objective magnification) were selected after pathological review, avoiding areas of necrosis, hemorrhage, large vessels, and tissue-processing artefacts. The same predefined regions were analyzed across all immunohistochemical stains to ensure spatial correspondence between markers. For this purpose, three representative photographs (×20 objective magnification) from each stain and each compartment were taken using the Nikon Digital Sight DS-Fi1 (5M2) camera and the Nikon Digital Sight DS-L2 imaging controller. The numbers of T8 lymphocytes (CD8^+^), CD68^+^ macrophages, CD163^+^ macrophages, and CD34^+^ microvessels were counted manually. The density of positive cells in each tumor compartment was assessed as the ratio of cell counts per mm^2^ surface. The complete immunohistochemical quantification was independently performed by two experienced hepatopathologists, who were blinded to the patients’ clinical outcomes and to each other’s measurements. Inter-observer agreement was assessed from the paired quantitative measurements independently obtained by the two hepatopathologists across the complete evaluated dataset using the intraclass correlation coefficient (ICC), with a corresponding 95% confidence interval.

### 2.5 Double immunofluorescence staining

Double immunofluorescence staining was performed on FFPE whole-mount tissue sections to characterize tumor-associated neutrophils and evaluate compartment-specific CD177 expression. Tissue sections (4 μm) were deparaffinized, rehydrated, and subjected to heat-induced antigen retrieval using Target Retrieval Solution, High pH (Dako Omnis; Agilent Technologies, Santa Clara, CA, USA). Following blocking of non-specific binding, sections were incubated overnight at 4 °C with primary antibodies against neutrophil elastase (NE; 1:200, HPA066836, Atlas Antibodies) and CD177 (1:100, clone C-5, sc-376329, Santa Cruz Biotechnology). Following incubation with appropriate fluorescence-conjugated secondary antibodies, m-IgG Fc BP-CFL 488 (1/200; sc-533653; Santa Cruz Biotechnology) and Drop-n-Stain CF594 goat anti-rabbit IgG (H+L), highly cross-adsorbed (Biotium), nuclei were counterstained with DAPI, and sections were mounted for imaging. Negative controls were processed in parallel by omission of the primary antibodies and potential cross-reactivity between the detection reagents was minimized through species-specific labeling (**Supplementary Table S1**).

### 2.6 Immunofluorescence analysis

Slides were digitally scanned at ×20 magnification using an Olympus SLIDEVIEW VS200 slide scanner (Olympus, Tokyo, Japan), and image analysis was performed using QuPath software (version 0.7.0) (27). Whole-slide images and the resulting quantitative measurements were independently evaluated by two experienced hepatopathologists blinded to the patients’ clinical outcomes and to each other’s measurements. For each tissue compartment, ten non-overlapping hotspot high-power fields (HPFs), corresponding to an aggregate analyzed area of approximately 1 mm², were selected from regions with the highest density of NE^+^ neutrophilic infiltration. Hotspot selection was guided by the corresponding serial hematoxylin-and-eosin whole-mount sections to confirm tissue architecture and neutrophilic infiltration. Areas containing necrosis, hemorrhage, large vessels, or processing artefacts were excluded.

Quantification of the studied markers was performed using optimized automated detection algorithms in QuPath, followed by independent manual verification by both hepatopathologists. For double immunofluorescence, neutrophils were identified as NE^+^ cells, whereas CD177^+^ neutrophils were defined as NE-positive cells demonstrating co-localized CD177 fluorescence. Neutrophils located within tumor area or hepatic parenchyma and sinusoidal spaces were included in the analysis, while cells located within large vascular lumina were excluded. For each patient and tissue compartment, measurements from ten HPFs were averaged to generate patient-level values. Patient-level outcomes included total NE^+^ density, absolute CD177^+^NE^+^ density, the CD177^+^ fraction of NE^+^ neutrophils, and estimated NE^+^CD177^−^ density. Estimated NE^+^CD177^−^ density was calculated for each patient-compartment pair by subtracting CD177^+^NE^+^ density from total NE^+^ density, and it was not measured as an independently stained population. These immunofluorescence hotspots were selected independently of the representative fields used for immunohistochemical quantification.

### 2.7 Primary and secondary outcomes

The primary outcome was the compartment-dependent distribution of tumor-associated neutrophils across the tumor center (TC), tumor margin/interface (IM), and adjacent non-neoplastic liver (NNL). This was assessed using NE^+^ density, CD177^+^NE^+^ density, the CD177^+^fraction of NE^+^ neutrophils, and estimated NE^+^CD177^−^ density. The primary objective was to determine whether tumor-associated neutrophil accumulation is accompanied by spatial differences in the relative representation of CD177-defined neutrophil populations. Additional analyses examined associations between tissue immune phenotypes and clinicopathological characteristics, systemic inflammatory indices, and other components of the immune and vascular microenvironment. Emphasis was placed on the relationship between preoperative neutrophil-to-lymphocyte ratio (NLR) and compartment-specific neutrophil phenotypes and CD163/CD68 macrophage polarization. Postoperative recurrence and survival were not included among the outcomes of the present spatial immunophenotyping study.

### 2.8 Statistical analysis

All analyses were performed using R version 4.5.2 (R Foundation for Statistical Computing, Vienna, Austria), with the patient considered the biological unit of replication. Image-level measurements were aggregated within each patient-compartment pair before analysis to preserve the paired design and avoid pseudoreplication. Continuous variables are presented as median and interquartile range (IQR), and categorical variables as frequencies and percentages. Compartmental differences across TC, IM, and NNL were assessed using the Friedman test in patients with complete triplets, with Kendall’s W reported as effect size. Significant overall tests were followed by paired Wilcoxon signed-rank tests with Holm correction. To incorporate all available observations and account for within-patient correlation, complementary generalized linear mixed-effects models (GLMMs) were fitted with tissue compartment as a fixed effect and patient as a random intercept. Negative-binomial models with analyzed tissue area as an offset were used for neutrophil counts, whereas the CD177-positive fraction was analyzed using a beta-binomial mixed-effects model based on CD177^+^NE^+^ and estimated NE^+^CD177^−^ counts. Immunohistochemical markers were additionally evaluated using mixed-effects models of log1p-transformed patient-level values. Model-derived pairwise contrasts were Holm-adjusted.

Four predefined patient-level neutrophil remodeling indices were calculated: log_2_ NE^+^ enrichment in TC relative to NNL, log_2_ NE^+^ enrichment in IM relative to NNL, and percentage-point CD177 loss from NNL to TC and from NNL to IM. One-sample Wilcoxon signed-rank tests assessed deviation from zero, with Holm correction across the four indices. Associations with continuous or ordinal clinicopathological and systemic inflammatory variables were assessed using Spearman’s rank correlation, while binary-group comparisons used Wilcoxon rank-sum tests with rank-biserial correlation as effect size. Exploratory associations between spatial-distribution indices and clinicopathological variables were evaluated using Spearman’s rank correlation or Wilcoxon rank-sum tests, as appropriate. P values for these clinicopathological comparisons were adjusted using the Holm procedure. For other predefined families of exploratory analyses, including associations with systemic inflammatory and tissue immune markers, the Benjamini–Hochberg (BH) false-discovery-rate procedure was applied, as specified for each analysis. To investigate the relationship between systemic inflammation and tissue myeloid organization, a CD163/CD68 macrophage-polarization index was calculated separately for each compartment and analyzed on the log_2_ scale. As CD163 and CD68 were assessed independently on separate tissue sections, the CD163/CD68 macrophage-polarization index was interpreted as a relative polarization measure rather than a cellular fraction, and it is an operational tissue-level abundance ratio. It does not represent the proportion of CD163-positive cells among CD68-positive cells and cannot demonstrate co-expression or macrophage polarization at single-cell resolution.

Preoperative NLR was analyzed both continuously and using a predefined clinically relevant threshold of 3 (NLR ≥3 vs <3), selected *a priori* based on previous evidence in HCC (28–30). Associations with continuous NLR were assessed using Spearman’s rank correlation, whereas comparisons between NLR groups used the Wilcoxon rank-sum test. Generalized estimating equations (GEE) incorporating compartment-by-NLR interaction terms were used to determine whether these associations differed across tissue compartments. Corresponding analyses of NE^+^ density, estimated NE^+^CD177^−^ density, and the CD177^+^neutrophil fraction assessed whether systemic inflammatory status was similarly associated with neutrophil abundance or phenotype.

All tests were two-sided, with *P <*0.05 considered statistically significant for primary analyses. Inter-observer agreement across the evaluated image measurements was assessed using the ICC, with a corresponding 95% confidence interval. Exploratory findings were interpreted according to effect sizes, confidence intervals, and false-discovery-rate-adjusted q values rather than nominal P values alone. Missing data were handled by complete-case analysis without imputation. The analysis workflow used the following R packages: readxl, dplyr, tidyr, stringr, purrr, readr, tibble, ggplot2, scales, glmmTMB, emmeans, and patchwork. The corresponding CRAN versions were readxl 1.5.0.1, dplyr 1.2.1, tidyr 1.3.2, stringr 1.6.0, purrr 1.2.2, readr 2.2.0, tibble 3.3.1, ggplot2 4.0.3, scales 1.4.0, glmmTMB 1.1.14, emmeans 2.0.4, and patchwork 1.3.2.

## 3 Results

### 3.1 Patient characteristics

The final spatial immunophenotyping cohort comprised 28 patients with HCC (**Table 1**). Twenty-three patients (82.1%) were male, and the median age at surgery was 60.5 years (IQR 52.0–66.0). Curative-intent surgical treatment consisted of liver transplantation in 18 patients (64.3%) and hepatic resection in 10 patients (35.7%). HBV without another listed etiology was present in 15 patients (53.6%), including one patient with HBV/HDV coinfection. One patient (3.6%) had HBV with alcohol-related liver disease, one (3.6%) had HBV with autoimmune hepatitis, two (7.1%) had HCV, four (14.3%) had alcohol-related liver disease without HBV, and two (7.1%) had MASH-related liver disease. No underlying liver-disease etiology was documented in three patients (10.7%). Child–Pugh classes A, B, and C were present in 19 (67.9%), seven (25.0%), and two (7.1%) patients, respectively. The median Child–Pugh score was 6.0 (IQR 5.0–8.0), and the median MELD score was 8.52 (IQR 7.04–12.99). Median preoperative AFP was 6.85 ng/mL (IQR 2.92–30.43). Median platelet, absolute lymphocyte, and absolute neutrophil counts were 119.5 × 10^9^/L (IQR 88.3–171.0), 1.55 × 10^9^/L (IQR 1.07–1.85), and 3.75 × 10^9^/L (IQR 3.08–4.70), respectively. The median preoperative NLR was 2.68 (IQR 1.60–3.75).

**Table 1:** Clinicopathological characteristics of the analysis cohort.

Characteristic	Value
Patients, n	28
Male sex, n (%)	23 (82.1%)
Age at surgery, median (IQR), years	60.50 (52.00–66.00)
BMI, median (IQR), kg/m²	27.07 (24.21–29.40)
HBV*	15 (53.6)
HBV and alcohol-related liver disease	1 (3.6)
HBV and autoimmune hepatitis	1 (3.6)
HCV	2 (7.1)
Alcohol-related liver disease	4 (14.3)
MASH	2 (7.1)
Other etiology	3 (10.7)
High grade, n (%)	4 (14.8%)
Portal-vein thrombosis at diagnosis, n (%)	2 (7.1%)
Pathological nodal involvement, n (%)	1 (3.6%)
Child–Pugh class A	19 (67.9%)
Child–Pugh class B	7 (25.0%)
Child–Pugh class C	2 (7.1%)
Child–Pugh score, median [IQR]	6.0 [5.0–8.0]
MELD score, median [IQR]	8.52 [7.04–12.99]
AFP, median [IQR]	6.85 [2.92–30.43]
Platelet count, ×10^9^/L, median [IQR]	119.5 [88.3–171.0]
Absolute lymphocyte count, ×10^9^/L, median [IQR]	1.55 [1.07–1.85]
Absolute neutrophil count, ×10^9^/L, median [IQR]	3.75 [3.08–4.70]
NLR, median [IQR]	2.68 [1.60–3.75]
Liver transplantation, n (%)	18 (64.3%)
Transplantation within Milan criteria at diagnosis	11 (61.1%)
Transplantation beyond Milan criteria at diagnosis	7 (38.9%)
Liver resection, n (%)	10 (35.7%)
Any locoregional treatment	8 (28.6%)
Transarterial chemoembolization	7 (25.0%)
Radiofrequency ablation	3 (10.7%)
Previous hepatic resection	2 (7.1%)
Time from HCC diagnosis to transplantation, months, median‡	15.3 [7.3–24.3]

Values are shown as n (%) or median (IQR), as indicated. Percentages are calculated using the total number of evaluable patients unless otherwise specified; grade percentages are calculated among patients with available grade data. The cohort includes 28 evaluable patients after exclusion of non-informative tissue material. *Including one HBV/HDV coinfection. IQR, interquartile range; AFP, alpha-fetoprotein; HBV, hepatitis B virus; HCC, hepatocellular carcinoma; HCV, hepatitis C virus; HDV, hepatitis D virus; IQR, interquartile range; MASH, metabolic dysfunction-associated steatohepatitis; MELD, Model for End-Stage Liver Disease; BCLC, Barcelona Clinic Liver Cancer; BMI, body mass index; NLR, neutrophil-to-lymphocyte ratio.

Eight patients (28.6%) had received prior locoregional treatment. Seven patients (25.0%) received TACE and three (10.7%) underwent RFA, with some patients receiving more than one treatment modality; none had received radiotherapy. Two patients (7.1%) had undergone previous hepatic resection. Among the 17 transplant recipients for whom both diagnosis and transplantation dates were available, the median interval from HCC diagnosis to transplantation was 15.3 months (IQR 7.3–24.3). Based on pretreatment clinical characteristics, 20 patients (71.4%) were classified as BCLC stage 0/A and eight (28.6%) as BCLC stage B; no patient had pretreatment BCLC stage C or D disease. No tumor vascular invasion was recorded. Portal-vein thrombosis was documented separately in two patients (7.1%) and one patient (3.6%) had nodal involvement identified on postoperative pathological examination following curative-intent hepatic resection. Among the 18 transplant recipients, 11 (61.1%) were within Milan criteria at diagnosis and seven (38.9%) were beyond Milan criteria. Five of the seven beyond-Milan patients received locoregional treatment before transplantation. No transplant recipient had an AFP concentration above 1,000 ng/mL. All 10 patients who underwent hepatic resection had Child–Pugh class A liver function and resectable disease. The median tumor diameter was 3.88 cm (IQR 2.95–4.62), and multifocal disease was present in 10 patients (35.7%). Histologically, the median tumor grade was 2 (IQR 2–2), indicating that most tumors were moderately differentiated. Overall inter-observer agreement across the evaluated image measurements was good (ICC = 0.84, 95% CI 0.72–0.89).

### 3.2 Tumor-associated compartments show neutrophil enrichment and distinct compartmental patterns of CD177-defined populations

Immunofluorescence staining using NE and CD177 demonstrated distinct spatial distributions of total NE^+^ and CD177^+^NE^+^ neutrophil populations across the TC, IM, and adjacent NNL (**Figure 1**). Quantitative analysis confirmed marked compartment-dependent neutrophil accumulation, together with substantial differences in the relative representation of CD177-defined neutrophil populations across tissue compartments (**Figure 2**). Total NE^+^ neutrophil density differed significantly across compartments (Friedman *P* < 0.001; Kendall’s W = 0.55), with median densities of 46.40 cells/mm² (IQR 19.05–101.95) in the TC, 39.65 cells/mm² (IQR 29.52–49.38) at the IM, and 8.80 cells/mm² (IQR 5.30–13.97) in adjacent NNL.

**Figure 1:**
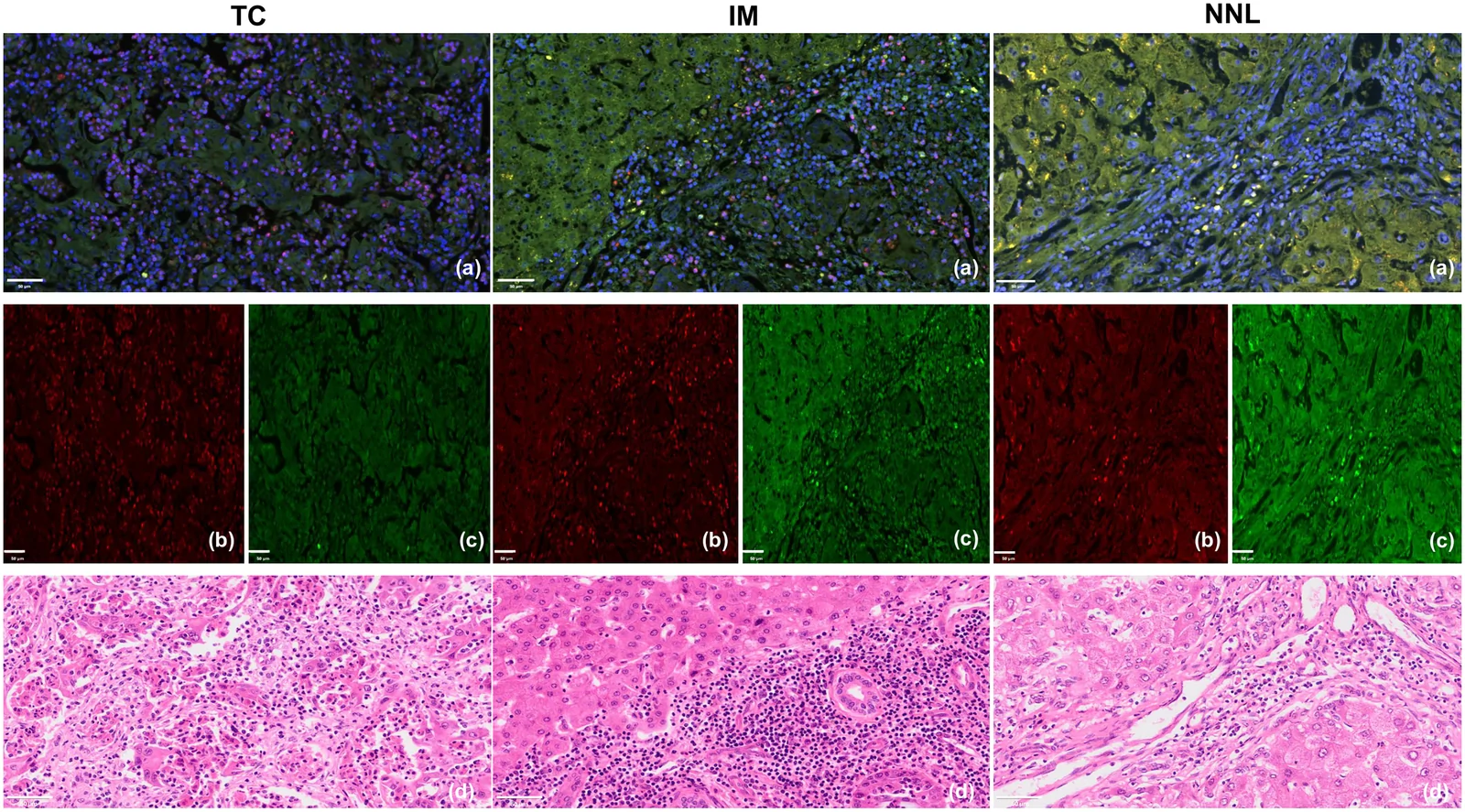
Representative immunofluorescence staining demonstrates compartment-specific neutrophil patterns across the hepatocellular carcinoma microenvironment. Representative fields from the tumor center (TC), tumor margin/interface (IM), and adjacent non-neoplastic liver (NNL) are shown. All illustrated fields were obtained from the same representative patient and were selected following pathological review to illustrate the characteristic staining patterns in each tissue compartment. For each compartment, panels **(a-c)** represent different channel views of the same immunofluorescence field: **(a)** merged DAPI (blue), neutrophil elastase (NE; red), and CD177 (green); **(b)** the corresponding NE channel; and **(c)** the corresponding CD177 channel. Panel **(d)** shows the corresponding area on the serial hematoxylin-and-eosin-stained section. One representative immunofluorescence field and one corresponding H&E field are shown for each compartment, comprising three immunofluorescence fields and three corresponding H&E fields in total. Slides were scanned at ×20 original magnification. Scale bars = 50 μm in every panel.

**Figure 2:**
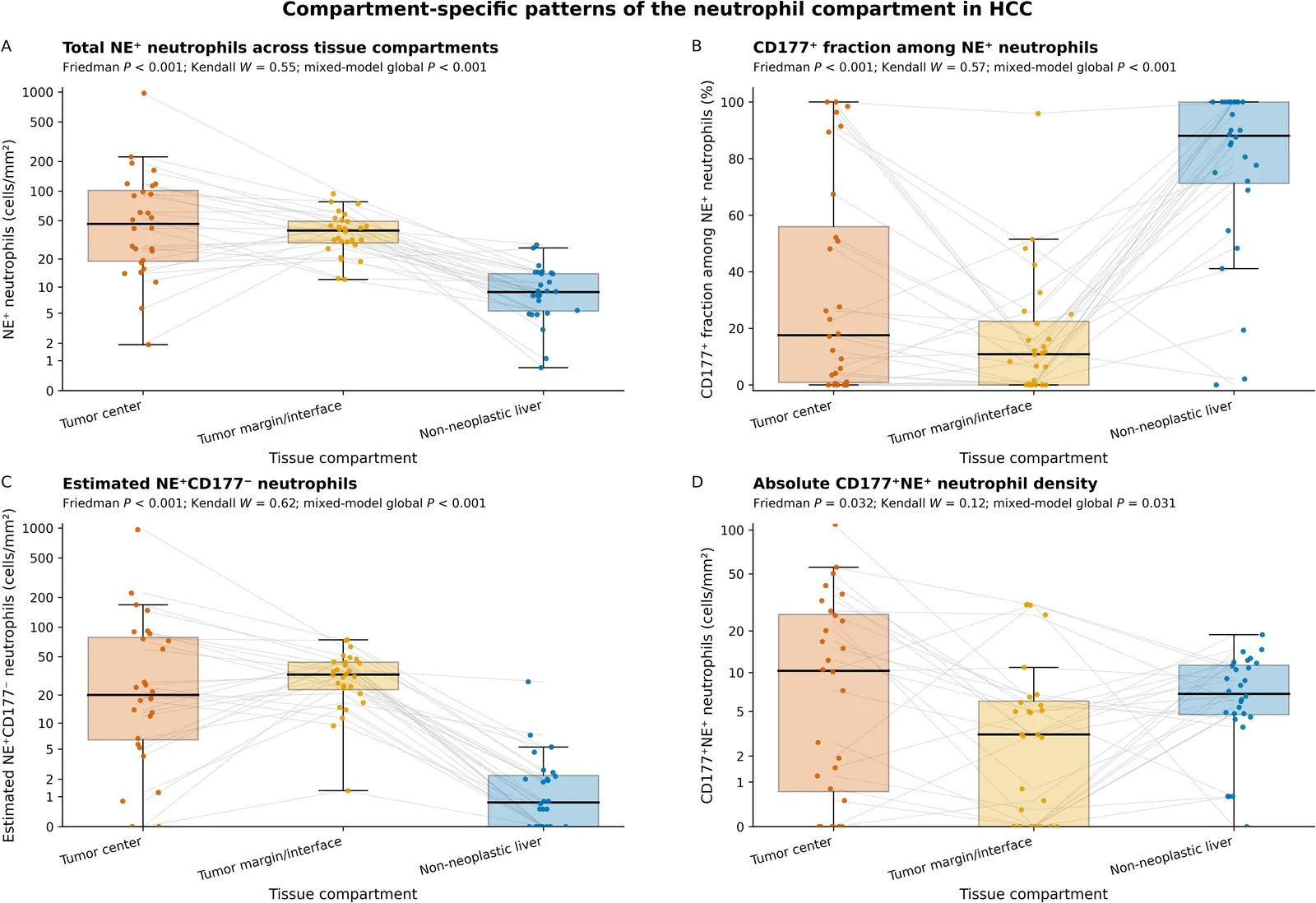
Spatial distribution of CD177-defined neutrophil populations across hepatocellular carcinoma tissue compartments. **(A)** Total neutrophil elastase-positive (NE^+^) neutrophil density, **(B)** proportion of CD177^+^ neutrophils among total NE^+^ neutrophils, **(C)** estimated density of CD177^-^ neutrophils (calculated as total NE^+^ minus CD177^+^NE+ neutrophils), and **(D)** CD177^+^NE^+^ neutrophil density in the tumor center (TC), tumor margin/interface (IM), and adjacent non-neoplastic liver (NNL). Each point represents an individual patient, with gray connecting lines indicating paired measurements across tissue compartments. Boxplots display the median and interquartile range (IQR), with whiskers representing 1.5× IQR. Overall compartmental differences were assessed using the Friedman test, with effect sizes reported as Kendall’s coefficient of concordance (Kendall’s W). Pairwise comparisons and global mixed-effects model P values are indicated within the panels. TC, tumor center; IM, tumor margin/interface; NNL, non-neoplastic liver; NE, neutrophil elastase.

Pairwise comparisons confirmed significantly higher densities in both tumor-associated compartments than in NNL (TC vs NNL: median paired difference 39.40 cells/mm², Holm-adjusted *P* < 0.001; IM vs NNL: median paired difference 23.60 cells/mm², Holm-adjusted *P* < 0.001), with density also significantly higher in the TC than at the IM (Holm-adjusted *P* = 0.044). Negative-binomial mixed-effects modeling confirmed a strong compartment effect (likelihood-ratio χ² = 58.64, *P* < 0.001). Relative to NNL, estimated neutrophil density was 7.07-fold higher in the TC (RR 7.07, 95% CI 4.60–10.88; Holm-adjusted *P* < 0.001) and 3.88-fold higher at the IM (RR 3.88, 95% CI 2.62–5.75; Holm-adjusted *P* < 0.001), and was also higher in the TC than at the IM (RR 1.82, 95% CI 1.21–2.75; Holm-adjusted *P* = 0.004). Predicted densities were 73.19 cells/mm² (95% CI 52.10–102.81), 40.13 cells/mm² (95% CI 29.49–54.61), and 10.34 cells/mm² (95% CI 7.55–14.17) in the TC, IM, and NNL, respectively (**Figure 3**).

**Figure 3:**
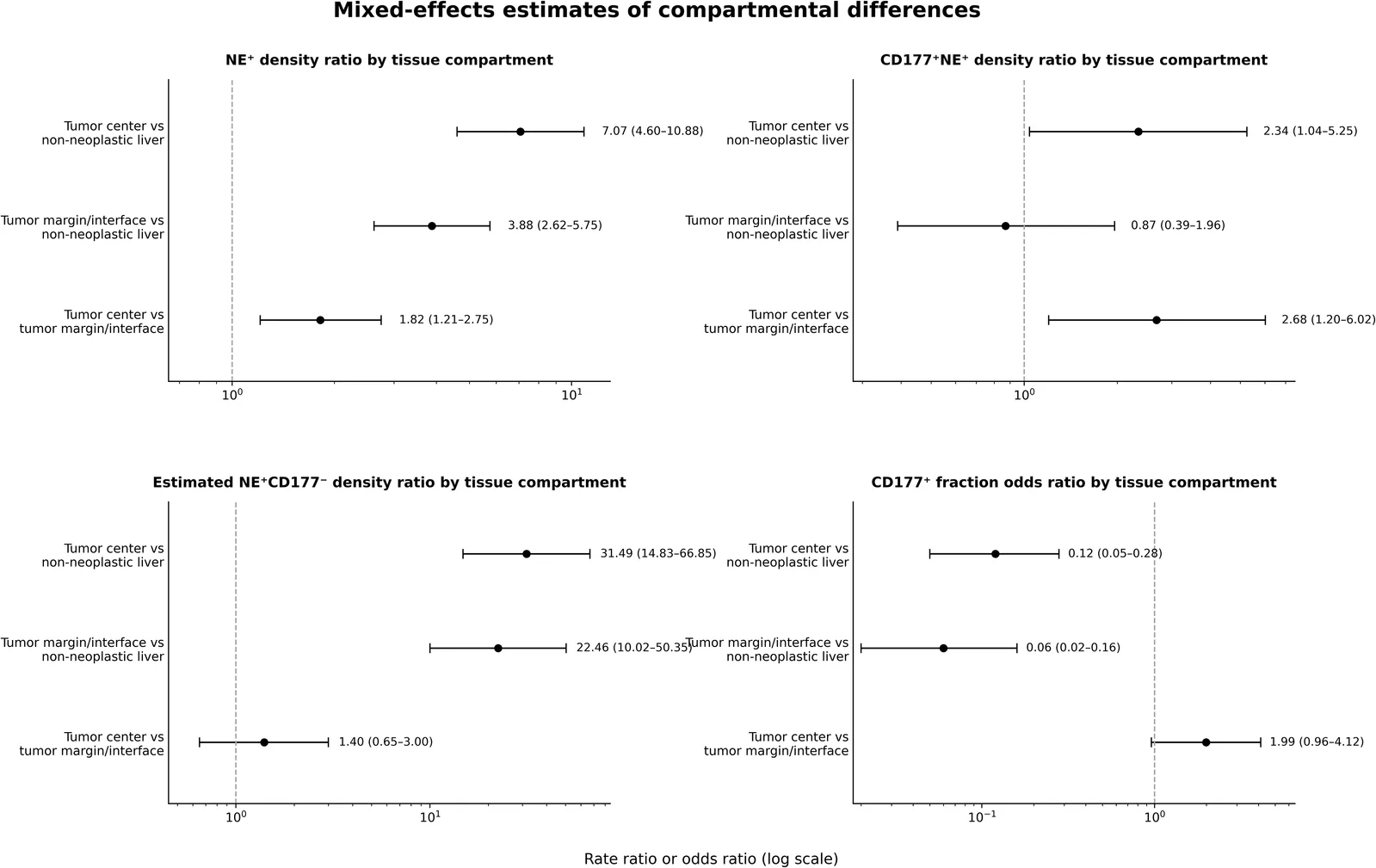
Mixed-effects modeling confirms compartment-dependent differences in neutrophil distribution. Forest plots showing estimated pairwise differences between tissue compartments derived from generalized linear mixed-effects models with patient included as a random intercept. Panels show comparisons for **(A)** total NE^+^ neutrophil density, **(B)** CD177^+^NE^+^ neutrophil density, **(C)** estimated CD177^-^ neutrophil density, and **(D)** the CD177^+^ fraction among NE^+^ neutrophils. Count outcomes are presented as rate ratios, whereas the CD177^+^ fraction is presented as odds ratios. Error bars indicate 95% confidence intervals. Pairwise comparisons were adjusted using the Holm procedure. The vertical dashed line represents the null value (rate ratio or odds ratio = 1). NE, neutrophil elastase.

Patient-level analyses confirmed consistent tumor-associated neutrophil enrichment. Median log_2_ enrichment relative to matched NNL was 2.15 (IQR 0.93–3.97) in the TC and 1.91 (IQR 1.35–2.72) at the IM, corresponding to approximately fourfold and 3.8-fold increases in neutrophil density, respectively (**Figure 4**). Both indices differed significantly from zero (Holm-adjusted *P* < 0.001). Despite this overall enrichment, CD177-defined neutrophil populations showed a distinct spatial distribution. CD177^+^NE^+^ density differed across compartments (Friedman *P* = 0.032; Kendall’s W = 0.12), although the overall effect size was small. Median densities were 10.30 cells/mm² (IQR 0.73–26.18) in the TC, 3.20 cells/mm² (IQR 0.00–6.05) at the IM, and 6.90 cells/mm² (IQR 4.72–11.32) in NNL. Pairwise analysis showed higher CD177^+^NE^+^ density in the TC than at the IM (Holm-adjusted *P* = 0.028), whereas neither TC nor IM differed significantly from NNL (both Holm-adjusted *P* = 0.266). Mixed-effects modeling similarly identified a modest overall compartment effect (likelihood-ratio χ² = 6.92, *P* = 0.031), with significantly higher CD177^+^NE^+^ density in the TC than at the IM (RR 2.68, 95% CI 1.20–6.02; Holm-adjusted *P* = 0.050). TC versus NNL (RR 2.34, 95% CI 1.04–5.25; Holm-adjusted *P* = 0.078) and IM versus NNL (RR 0.87, 95% CI 0.39–1.96; Holm-adjusted *P* = 0.741) were not significant. Model-estimated densities were 17.95 cells/mm² (95% CI 10.15–31.75), 6.69 cells/mm² (95% CI 3.78–11.85), and 7.67 cells/mm² (95% CI 4.33–13.58) in the TC, IM, and NNL, respectively.

**Figure 4:**
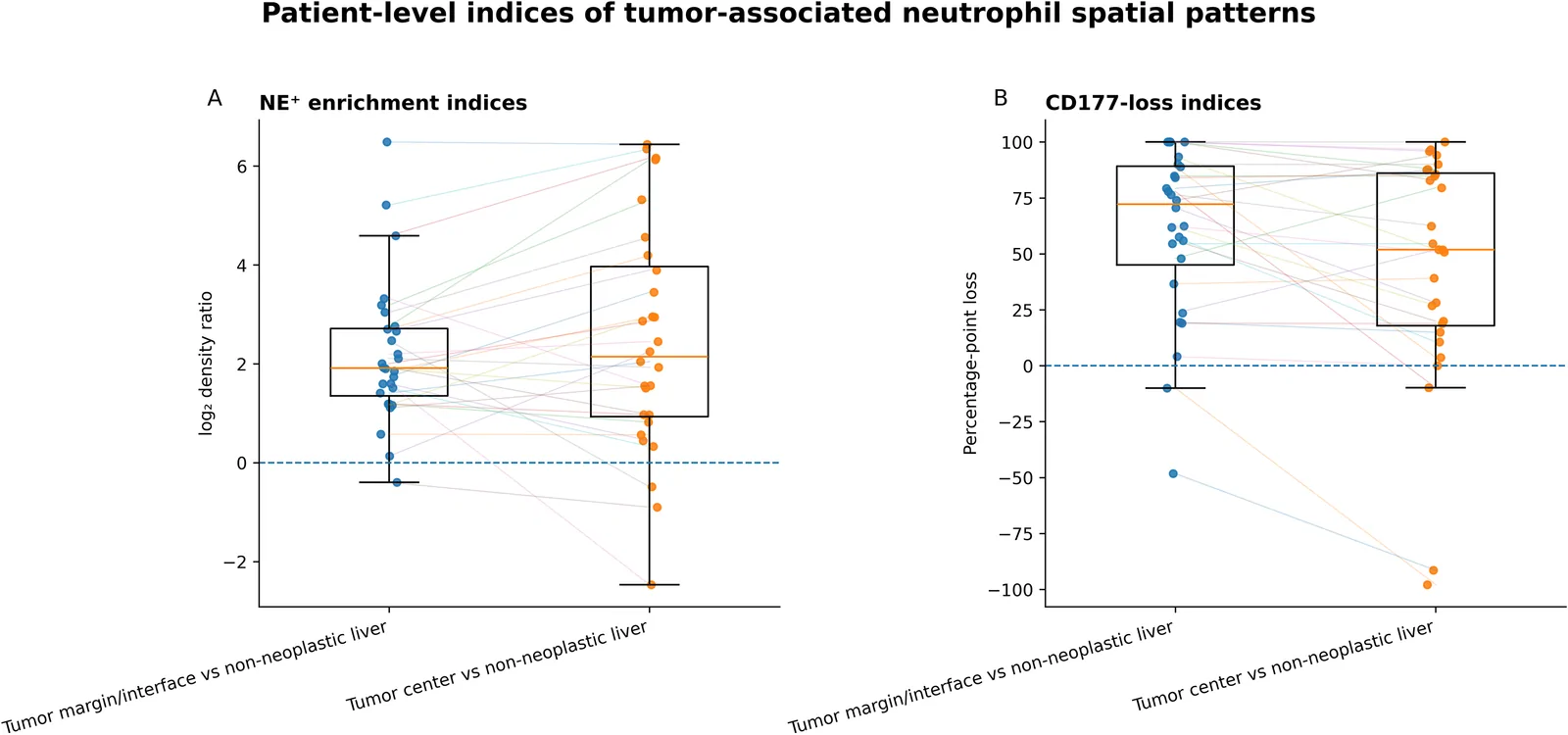
Patient-level spatial-distribution indices summarizing tumor-associated differences in neutrophil abundance and CD177-defined composition relative to adjacent non-neoplastic liver. **(A)** Log_2_ enrichment of total NE+ neutrophil density at the tumor margin/interface (IM/NNL) and tumor center (TC/NNL). **(B)** Percentage-point reduction in the CD177^+^ neutrophil fraction between non-neoplastic liver and the corresponding tumor compartment. Each point represents an individual patient, with gray lines connecting paired spatial-distribution indices. Boxplots display the median and interquartile range (IQR). The horizontal dashed line indicates the null value (no enrichment or no reduction in the CD177^+^ fraction). Statistical significance was assessed using one-sample Wilcoxon signed-rank tests with Holm correction for multiple comparisons. NE, neutrophil elastase; TC, tumor center; IM, tumor margin/interface; NNL, non-neoplastic liver.

In contrast, the CD177^+^ fraction of total NE^+^ neutrophils showed a pronounced compartmental difference (Friedman *P* < 0.001; Kendall’s W = 0.57). Median CD177 positivity decreased from 88.08% (IQR 71.23–100.00) in NNL to 17.63% (IQR 0.95–55.96) in the TC and 10.90% (IQR 0.00–22.50) at the IM. The CD177^+^ fraction was significantly lower in both the TC (median paired difference −51.86 percentage points; Holm-adjusted *P* = 0.002) and IM (−72.26 percentage points; Holm-adjusted *P* < 0.001) compared with NNL and remained higher in the TC than at the IM (+4.32 percentage points; Holm-adjusted *P* = 0.007). Beta-binomial mixed-effects modeling confirmed this pattern (likelihood-ratio χ²=39.16, *P* < 0.001) (**Figure 3**). Compared with NNL, the odds of CD177 expression were reduced by approximately 88% in the TC (OR 0.12, 95% CI 0.05–0.28; Holm-adjusted *P* < 0.001) and 94% at the IM (OR 0.06, 95% CI 0.02–0.16; Holm-adjusted *P* < 0.001), with no significant TC–IM difference (OR 1.99, 95% CI 0.96–4.12; Holm-adjusted *P* = 0.065). Predicted CD177^+^ fractions were 79.35% (95% CI 66.08–88.34) in NNL, 32.22% (95% CI 21.22–45.63) in the TC, and 19.32% (95% CI 11.35–30.92) at the IM.

Consistent with the lower CD177^+^ fraction in tumor-associated compartments, estimated NE^+^CD177^−^ neutrophils showed pronounced spatial enrichment (Friedman χ² = 34.55, *P* < 0.001; Kendall’s W = 0.62) (**Figures 2**, **3**). Median density increased from 0.75 cells/mm² (IQR 0.00–2.28) in NNL to 20.10 cells/mm² (IQR 6.45–78.55) in the TC and 32.75 cells/mm² (IQR 22.80–43.92) at the IM. Mixed-effects modeling confirmed substantially higher densities in the TC (RR 31.49, 95% CI 14.83–66.85; Holm-adjusted *P* < 0.001) and IM (RR 22.46, 95% CI 10.02–50.35; Holm-adjusted *P* < 0.001) compared with NNL, with no significant TC–IM difference (RR 1.40, 95% CI 0.65–3.00; Holm-adjusted *P* = 0.384). Model-estimated densities were 51.48 cells/mm² (95% CI 27.69–95.69) in the TC, 36.72 cells/mm² (95% CI 21.00–64.20) at the IM, and 1.64 cells/mm² (95% CI 0.85–3.16) in NNL.

### 3.3 Macrophage, lymphoid, and vascular populations show distinct spatial organization across HCC compartments

The broader immune and vascular microenvironment also demonstrated marked spatial organization (**Figure 5**; **Tables 2**, **3**; **Supplementary Figures S1**-**S3**). CD8^+^ lymphocytes differed significantly across compartments (Friedman *P* < 0.001; Kendall’s W = 0.30), with the highest median density at the IM (21.83 cells/mm^2^, IQR 12.25–33.17), compared with the TC (7.17, IQR 5.17–14.17) and NNL (8.50, IQR 7.50–16.75). Pairwise analyses confirmed significantly greater CD8^+^ infiltration at the IM than in both the TC and NNL after Holm correction, whereas TC and NNL did not differ significantly. Mixed-effects modeling reproduced this distribution, supporting preferential localization of cytotoxic T lymphocytes at the tumor-host interface.

**Figure 5:**
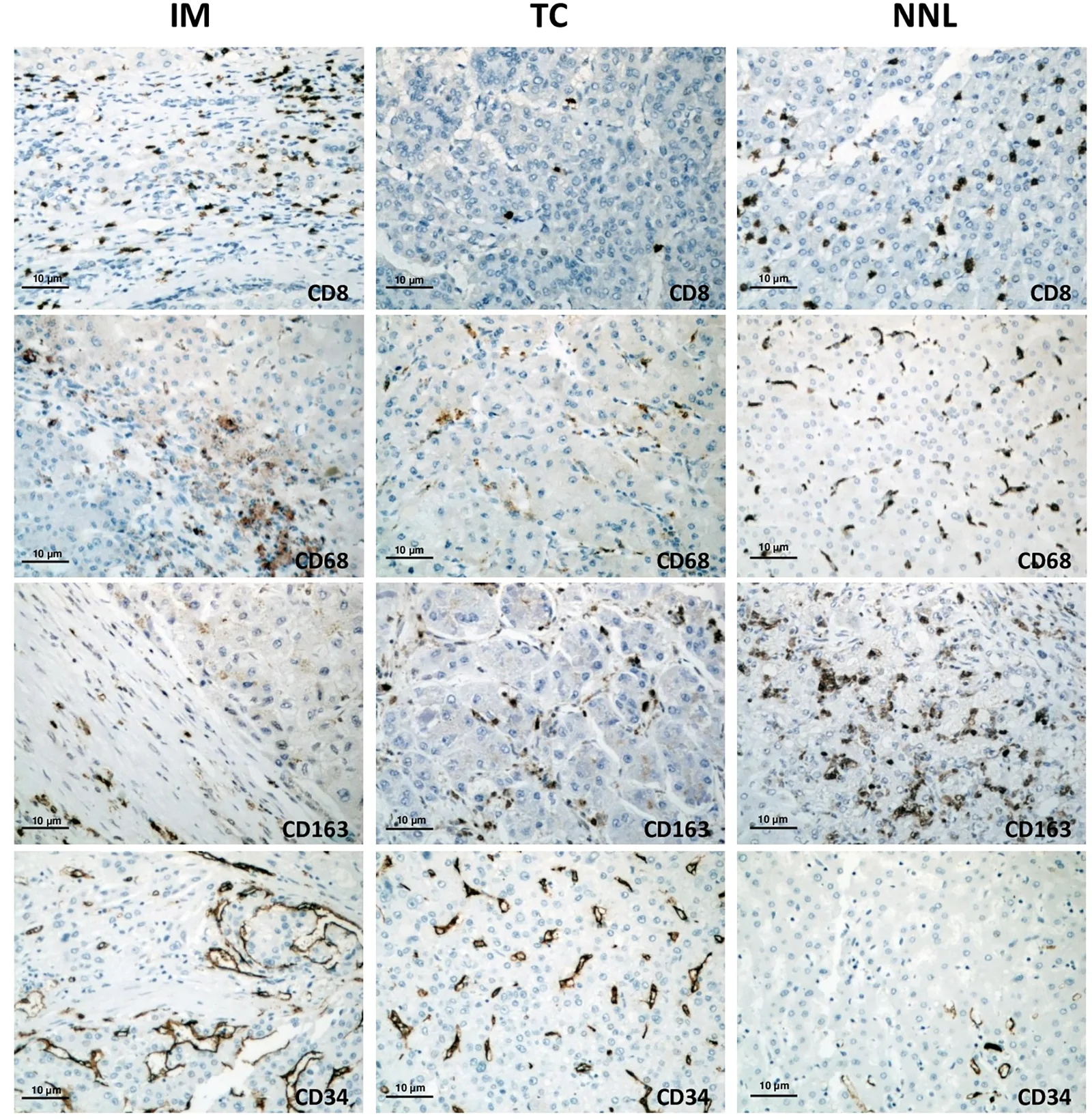
Representative immunohistochemical staining illustrating the spatial organization of the hepatocellular carcinoma immune microenvironment. Representative immunohistochemical fields demonstrate CD8^+^ cytotoxic T lymphocytes, CD68^+^ macrophages, CD163^+^ macrophages, and CD34^+^ microvessels across the tumor center (TC), tumor margin/interface (IM), and adjacent non-neoplastic liver (NNL). All illustrated fields were obtained from the same representative patient. The fields were selected following pathological review to illustrate the characteristic staining patterns in each compartment. One representative field is shown for each marker-compartment combination, comprising 12 fields in total. Images were acquired at ×20 objective magnification. Scale bars = 10 μm in every panel.

**Table 2:** Compartmental distribution of immune, angiogenic and neutrophil markers.

Marker/metric	Complete paired cases	Tumor center, median (IQR)	Tumor margin/interface, median (IQR)	Non-neoplastic liver, median (IQR)	Friedman *P*	Kendall W	Brief interpretation
CD8^+^ T cells, cells/mm^2^	28	7.17 (5.17–14.17)	21.83 (12.25–33.17)	8.50 (7.50–16.75)	< 0.001	0.30	Higher at tumor margin/interface; compatible with interface-predominant T-cell infiltration.
CD68^+^ macrophages, cells/mm^2^	28	24.00 (12.83–33.25)	28.33 (20.25–38.33)	41.67 (34.17–49.00)	0.003	0.21	Higher in non-neoplastic liver; significant but modest compartmental effect.
CD163^+^ macrophages, cells/mm^2^	28	7.17 (4.25–11.08)	4.50 (2.33–6.67)	5.33 (3.33–8.08)	0.045	0.11	Small overall compartmental difference; mixed-effects modelling showed higher CD163^+^ density in the TC than at the IM.
CD34^+^ microvessels/angiogenesis, vessels/mm^2^	28	25.50 (16.58–34.42)	15.17 (11.17–24.08)	9.17 (6.58–14.67)	< 0.001	0.51	Highest in tumor center and lowest in non-neoplastic liver; strong angiogenesis-related compartmental pattern.
NE^+^ neutrophils, cells/mm²	28	46.40 (19.05–101.95)	39.65 (29.52-49.38)	8.80 (5.30–13.97)	< 0.001	0.55	NE^+^ neutrophils were enriched in tumor-associated compartments and lowest in non-neoplastic liver.
CD177^+^NE^+^ neutrophils, cells/mm²	28	10.30 (0.73–26.18)	3.20 (0.00–6.05)	6.90 (4.72–11.32)	0.032	0.12	Statistically significant overall compartmental difference in CD177^+^NE^+^ density
CD177^+^NE^+^ among NE+ neutrophils, %	28	17.63 (0.95–55.96)	10.90 (0.00–22.50)	88.08 (71.23–100.00)	< 0.001	0.57	CD177^+^ fraction was markedly higher in non-neoplastic liver and reduced near/in tumor tissue.

Values are patient-level compartment summaries expressed as median (IQR). IHC markers are reported as cells/mm^2^ or counts/mm^2^; immunofluorescence neutrophil markers are reported as cells/mm² or percentage, as indicated. The Friedman test evaluates the global paired difference across TC, IM and NNL. Kendall’s W is reported as an effect-size estimate, with larger values indicating stronger compartmental separation.

**Table 3:** Pairwise *post-hoc* comparisons between tissue compartments.

Marker	Comparison	n pairs	Median difference*	Raw *P*	Holm-adjusted *P*	Interpretation
CD8	Tumor center vs Tumor margin/interface	28	-9.67	< 0.001	< 0.001	Higher in Tumor margin/interface
CD8	Tumor center vs Non-neoplastic liver	28	-1.00	0.502	0.502	Not significant after Holm correction
CD8	Tumor margin/interface vs Non-neoplastic liver	28	10.00	< 0.001	0.002	Higher in Tumor margin/interface
CD68	Tumor center vs Tumor margin/interface	28	-3.00	0.316	0.316	Not significant after Holm correction
CD68	Tumor center vs Non-neoplastic liver	28	-15.50	0.004	0.008	Higher in Non-neoplastic liver
CD68	Tumor margin/interface vs Non-neoplastic liver	28	-11.83	< 0.001	< 0.001	Higher in Non-neoplastic liver
CD163	Tumor center vs Tumor margin/interface	28	2.00	0.017	0.052	Borderline after Holm correction
CD163	Tumor center vs Non-neoplastic liver	28	2.67	0.260	0.260	Not significant after Holm correction
CD163	Tumor margin/interface vs Non-neoplastic liver	28	-1.17	0.075	0.151	Not significant after Holm correction
CD34	Tumor center vs Tumor margin/interface	28	10.00	0.007	0.007	Higher in Tumor center
CD34	Tumor center vs Non-neoplastic liver	28	12.67	< 0.001	< 0.001	Higher in Tumor center
CD34	Tumor margin/interface vs Non-neoplastic liver	28	6.00	0.003	0.005	Higher in Tumor margin/interface
NE^+^	Tumor center vs Tumor margin/interface	28	10.90	0.044	0.044	Higher in Tumor center
NE^+^	Tumor center vs Non-neoplastic liver	28	39.40	< 0.001	< 0.001	Higher in Tumor center
NE^+^	Tumor margin/interface vs Non-neoplastic liver	28	23.60	< 0.001	< 0.001	Higher in Tumor margin/interface
CD177^+^NE^+^	Tumor center vs Tumor margin/interface	28	2.15	0.009	0.028	Higher in Tumor center
CD177^+^NE^+^	Tumor center vs Non-neoplastic liver	28	0.85	0.133	0.266	Not significant after Holm correction
CD177^+^NE^+^	Tumor margin/interface vs Non-neoplastic liver	28	-4.20	0.136	0.266	Not significant after Holm correction
CD177^+^NE^+^ fraction	Tumor center vs Tumor margin/interface	28	4.32	0.007	0.007	Higher in Tumor center
CD177^+^NE^+^ fraction	Tumor center vs Non-neoplastic liver	28	-51.86	< 0.001	0.002	Higher in Non-neoplastic liver
CD177^+^NE^+^ fraction	Tumor margin/interface vs Non-neoplastic liver	28	-72.26	< 0.001	< 0.001	Higher in Non-neoplastic liver

Pairwise comparisons were performed using paired Wilcoxon signed-rank tests. The median difference is calculated as the first compartment minus the second compartment in each comparison. Holm-adjusted p values correct the three pairwise comparisons performed within each marker. Interpretations are based on Holm-adjusted P < 0.05.

CD34^+^ microvascular density also differed markedly across compartments (Friedman *P* < 0.001; Kendall’s W = 0.51). Median density progressively decreased from the TC (25.50 vessels/mm², IQR 16.58–34.42) to the IM (15.17 vessels/mm², IQR 11.17–24.08) and NNL (9.17 vessels/mm², IQR 6.58–14.67). All pairwise comparisons remained significant after Holm adjustment, and mixed-effects modeling confirmed the TC>IM>NNL gradient.

CD68^+^ macrophages showed significant compartmental variation (Friedman *P* = 0.003; Kendall’s W = 0.21), with median densities highest in NNL (41.67 cells/mm², IQR 34.17–49.00), followed by the IM (28.33 cells/mm², IQR 20.25–38.33) and TC (24.00 cells/mm², IQR 12.83–33.25). NNL contained significantly more CD68^+^ macrophages than either tumor-associated compartment, whereas TC and IM did not differ significantly. CD163^+^ macrophages showed more modest spatial heterogeneity (Friedman *P* = 0.045; Kendall’s W = 0.11), with median densities of 7.17 cells/mm^2^ (IQR 4.25–11.08) in the TC, 4.50 cells/mm^2^ (IQR 2.33–6.67) at the IM, and 5.33 cells/mm^2^ (IQR 3.33–8.08) in NNL. The paired Wilcoxon TC-versus-IM contrast did not remain significant after Holm correction (Holm-adjusted *P* = 0.052), whereas complementary mixed-effects modeling supported a modest overall compartment effect (*P* = 0.037), primarily reflecting higher CD163^+^ signal in the TC than at the IM.

A patient-level CD163/CD68 macrophage-polarization index was significantly higher in the TC than at the IM (median 32.39% [IQR 15.43–51.67] vs 15.29% [IQR 7.73–29.66]; median paired difference +11.28 index units; paired Wilcoxon *P* = 0.009) (**Supplementary Table S2**). The log_2_-transformed ratio yielded consistent results (median −1.63 [IQR −2.70 to −0.95] vs −2.71 [IQR −3.69 to −1.76]; median paired difference +0.94 log_2_ units; *P* = 0.009). Because CD68 and CD163 were assessed on separate tissue sections, this index represents an IHC-derived macrophage-polarization measure rather than a direct cellular fraction.

### 3.4 Systemic inflammation is associated with intratumoral macrophage polarization but not neutrophil distribution

Four patient-level neutrophil spatial-distribution indices were derived from paired tumor and NNL measurements: log_2_ NE^+^ enrichment in the TC relative to NNL, log_2_ NE^+^ enrichment at the IM relative to NNL, and the reduction in the CD177^+^ neutrophil fraction in the TC and IM relative to NNL. All differed significantly from zero (all adjusted *P* < 0.001), confirming consistent tumor-associated neutrophil enrichment and compartmental differences in CD177-defined populations across the cohort. No index remained significantly associated with tumor size, histological grade, tumor multiplicity, BCLC stage, or treatment modality after multiple-testing correction (all q > 0.05) (**Figure 6**; **Supplementary Tables S3**, **S4**). None of the four neutrophil spatial-distribution indices was significantly associated with preoperative NLR after multiple-testing correction (all q > 0.05). Although several nominal correlations were observed between total NE+ density and macrophage or vascular markers, none remained significant after correction. CD177^+^NE^+^ density and the CD177^+^ fraction showed only weak correlations with CD8^+^, CD68^+^, CD163^+^, and CD34^+^ markers across compartments (all |ρ| ≤ 0.29, all nominal *P* > 0.14).

**Figure 6:**
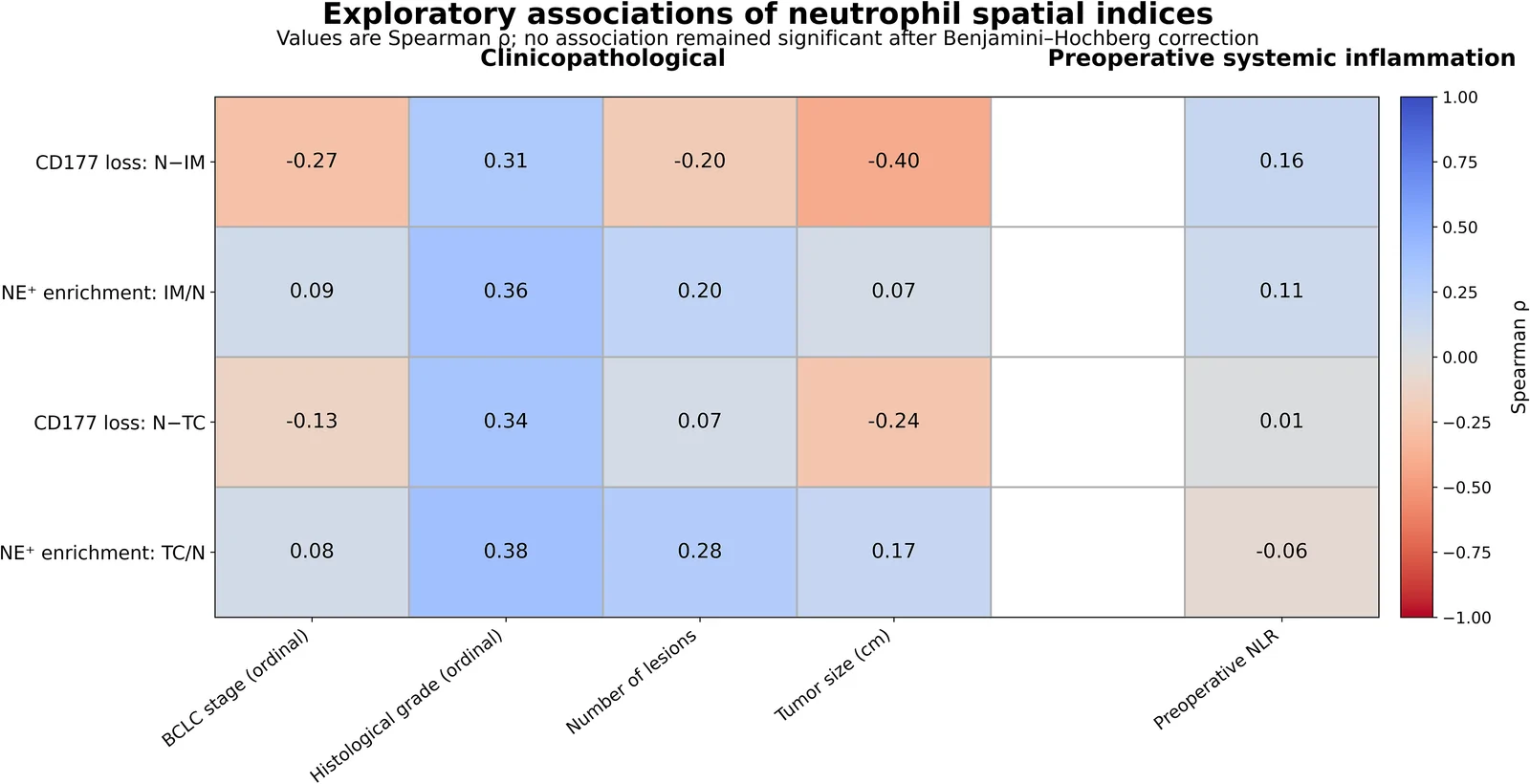
Associations of neutrophil-remodeling indices with clinicopathological characteristics and preoperative systemic inflammation. Heatmap summarizing exploratory associations between patient-level neutrophil-remodeling indices and BCLC stage, histological grade, number of lesions, tumor size, and preoperative peripheral-blood neutrophil-to-lymphocyte ratio (NLR). Tiles display Spearman’s rank correlation coefficients (ρ), with blue indicating positive and red indicating negative correlations. No association remained statistically significant after the prespecified multiple-testing corrections. BCLC, Barcelona Clinic Liver Cancer; NLR, neutrophil-to-lymphocyte ratio; TC, tumor center; IM, tumor margin/interface; NNL, non-neoplastic liver.

A different correlation was observed between macrophages and systemic inflammation. Preoperative NLR was positively associated with the compartment-specific CD163/CD68 macrophage-polarization index exclusively in the TC (Spearman’s ρ=0.451, *P* = 0.016; BH q = 0.048), with no significant association at the IM (ρ = −0.106, *P* = 0.591) or in NNL (ρ = 0.245, *P* = 0.209) (**Supplementary Table S5**; **Supplementary Figure S4**). Using the predefined NLR ≥ 3 threshold, patients with NLR≥3 had a higher TC CD163/CD68 index than those with NLR < 3 (median 49.1% vs 24.3%), although this difference did not remain significant after multiple-testing correction (BH q = 0.128); no differences were observed in the remaining compartments (**Supplementary Figure S5**; **Supplementary Table S5**).

Generalized estimating equations also indicated that the association between systemic NLR and the CD163/CD68 macrophage-polarization index differed by tissue compartment (**Supplementary Table S6**). A significant compartment-by-continuous-NLR interaction was observed (Wald χ² = 10.41, *P* = 0.005), with each one-standard-deviation increase in preoperative NLR associated with a 1.57-fold higher TC CD163/CD68 index (95% CI 1.20–2.07; *P* = 0.001), but no corresponding association at the IM or in NNL. Analysis using the NLR ≥ 3 threshold similarly demonstrated a significant compartment-by-group interaction (Wald χ² = 8.38, *P* = 0.015). Sensitivity analyses based on within-patient compartmental contrasts showed a corresponding association between higher systemic NLR and a higher CD163/CD68 macrophage-polarization index within the TC (**Supplementary Figure S6**).

In contrast, neutrophil spatial distribution was largely unrelated to systemic inflammatory status. Preoperative NLR was also unrelated to the CD177^+^ neutrophil fraction in the TC (ρ = 0.033, *P* = 0.866), IM (ρ = −0.132, *P* = 0.504), or NNL (ρ = 0.312, *P* = 0.106), with all adjusted q values >0.05 (**Supplementary Table S7**). Using the predefined NLR threshold of 3, the CD177^+^ fraction did not differ between patients with NLR ≥ 3 and NLR < 3 in any compartment. Median total NE^+^ densities were 41.8 versus 51.0 cells/mm² in the TC (*P* = 0.612), 43.8 versus 33.0 cells/mm² at the IM (*P* = 0.174), and 9.0 versus 8.5 cells/mm² in NNL (*P* = 0.596). Estimated NE^+^CD177^−^ density likewise showed no between-group differences in the TC (*P* = 0.890), IM (*P* = 0.167), or NNL (*P* = 0.944). Finally, no NLR-by-compartment interaction was detected for total NE^+^ density (*P* = 0.998) or estimated NE^+^CD177^−^ density (*P* = 0.978), indicating no detectable association between systemic inflammatory status and the spatial distribution of these neutrophil populations.

## 4 Discussion

The present study demonstrated three principal findings. First, HCC was characterized by marked spatial immune heterogeneity across TC, IM, and adjacent NNL, involving lymphoid, myeloid, and vascular components. Additionally, tumor-associated compartments contained higher total neutrophil densities and markedly lower CD177^+^ fractions than adjacent NNL. Finally, systemic inflammatory status showed distinct relationships with local myeloid populations: higher preoperative NLR was associated with increased CD163/CD68 macrophage polarization specifically within the TC, whereas neutrophil abundance and CD177-defined compartmental patterns were not significantly associated with NLR. These findings, which are observational and describe spatial differences and associations in marker-defined cell populations, revealed a spatially organized HCC immune microenvironment, where systemic inflammation uniquely links to distinct tissue myeloid compartments.

A sharp divergence was identified between neutrophil abundance and CD177 phenotype. Tumor-associated compartments were substantially enriched with total NE^+^ neutrophils, whereas CD177^+^NE^+^ density showed only a small overall compartmental effect, with higher density in the TC than at the IM. In contrast, the CD177^+^ fraction was markedly reduced in tumor-associated compartments, accompanied by substantial enrichment of estimated NE^+^CD177^−^ neutrophils. These findings are consistent with the increasingly recognized heterogeneity of TANs, which comprise multiple phenotypic and functional states shaped by local tissue signals rather than a homogeneous neutrophil population (7, 8, 31, 32). More specifically, single-cell evidence indicates that the neutrophil compartment in liver cancer differs between tumor tissue, adjacent liver, and peripheral blood. Xue et al. identified multiple tumor-associated neutrophil states in human liver cancer, including CCL4^+^ and PD-L1^+^ populations with experimentally supported macrophage-recruiting and T-cell-suppressive properties, respectively (33). These populations were particularly enriched in a myeloid-cell-dominant immunosuppressive tumor microenvironment subtype. Our finding of increased total NE^+^ neutrophil density accompanied by a markedly altered CD177-defined composition is consistent with the broader concept that neutrophil accumulation in HCC involves a change in population structure rather than uniform expansion of a homogeneous neutrophil population. However, the transcriptomic populations identified by Xue et al. cannot be mapped directly onto the CD177-defined populations measured in the present study. In particular, our data do not establish that the estimated NE^+^CD177⁻ population corresponds to the CCL4^+^, PD-L1^+^, or other functionally defined neutrophil states identified by single-cell sequencing.

The reduced CD177^+^ fraction within tumor-associated compartments should also be distinguished from an absolute loss of CD177^+^ neutrophils. CD177^+^NE^+^ density showed only a modest compartmental effect, whereas total NE^+^ density increased substantially. The lower CD177^+^ fraction may therefore reflect relative enrichment of other neutrophil populations within an expanded tumor-associated neutrophil compartment rather than selective depletion of CD177^+^ cells. Because the present study was cross-sectional, it cannot determine whether this pattern results from preferential recruitment, retention, differential survival, or phenotypic regulation.

Recent human evidence further indicates that CD177 expression may distinguish biologically stable neutrophil populations. Jung et al. reported that CD177⁻ neutrophils represented a stable human neutrophil subtype and were enriched in head-and-neck tumor tissue, where functional experiments indicated tumor-supporting properties (34). The relative enrichment of estimated NE^+^CD177⁻ neutrophils in our HCC specimens is compatible with tumor-associated selection of CD177⁻ populations. Nevertheless, the different tumor type and the absence of functional assays in our study preclude functional equivalence or classification of these cells as tumor-promoting. Conversely, tumor-suppressive, and favorable prognostic associations have been reported for CD177^+^ neutrophils in colitis-associated colorectal cancer (35), further emphasizing the context-dependent biology of CD177-defined populations.

Evidence from the human liver further illustrates the context-dependent biology of CD177-defined neutrophils. In patients undergoing associating liver partition and portal vein ligation for staged hepatectomy (ALPPS), Yan et al. combined single-cell and spatial profiling with functional experiments and identified an expansion of metabolically active CD177^+^ neutrophils that promoted endothelial transmigration through CD177-PECAM1 interactions and supported liver regeneration through MMP9-mediated extracellular-matrix remodeling and HGF-α release (36). This population also exhibited enhanced NET-forming capacity, while its transcriptional phenotype evolved from an early pro-inflammatory state toward a later pro-regenerative state. These observations support phase- and microenvironment-dependent functional plasticity rather than a simple abundance-dependent model. In particular, the study does not establish a numerical threshold at which a low abundance of CD177^+^ neutrophils is regenerative, whereas a high abundance becomes tissue damaging. Accordingly, the lower CD177^+^ fraction observed in the HCC-associated compartments in our study should not be interpreted as the inverse of the ALPPS response or as evidence of impaired regenerative activity. Instead, the contrast underscores that CD177 expression must be interpreted within the specific biological setting and that direct functional studies are required to determine whether CD177-defined neutrophils in HCC participate in tissue repair, inflammation, tumor support, or other context-dependent processes.

The broader immune landscape supported the spatial organization identified in HCC. CD8^+^ lymphocytes preferentially accumulated at the tumor-host interface, whereas CD34^+^ microvascular density followed a TC>IM>NNL gradient. Macrophage populations displayed a different pattern, with greater CD68^+^ density in NNL but a higher CD163/CD68 polarization index in the TC than at the IM. The higher CD68^+^ macrophage density in NNL likely reflects, at least in part, the physiological abundance of resident Kupffer cells in the NNL parenchyma. Accordingly, the more informative finding may be the relative macrophage composition within tumor-associated compartments, particularly the higher CD163/CD68 macrophage-polarization index in the TC compared with the IM. In the present study, CD68 and CD163 staining quantified relative tissue-level abundance and did not establish the functional behavior or pro-tumor versus anti-tumor activity of individual macrophages. These findings are also consistent with spatial studies demonstrating that human HCC contains anatomically distinct immune niches. Liu et al. identified a tumor-boundary immune barrier formed by SPP1^+^ macrophages and cancer-associated fibroblasts that restricted immune-cell penetration into the tumor core, whereas Wang et al. demonstrated heterogeneous distributions of lymphoid and myeloid populations across HCC regions (37, 38). The preferential accumulation of CD8^+^ lymphocytes at the IM and the compartment-specific differences in macrophage and vascular indices observed in our study accord with this broader spatial organization. However, differences in tissue segmentation, marker panels, treatment setting, and analytical resolution preclude direct equivalence between the cell populations identified by spatial transcriptomics and those defined using conventional immunohistochemistry in the present study. Tumor-associated macrophages are themselves highly heterogeneous and can acquire immunoregulatory and tumor-supporting states in response to hypoxia, tumor-derived cytokines, metabolic signals, and interactions with endothelial and stromal cells (19). Thus, the spatial differences observed across neutrophil, macrophage, lymphoid, and vascular compartments support a model in which TC and IM represent biologically distinct immune niches rather than different sampling regions of a homogeneous tumor microenvironment. Importantly, however, the limited correlations between CD177-defined neutrophil populations and individual CD8, macrophage, or vascular markers suggest that neutrophil remodeling cannot be attributed to any single measured component of this broader microenvironment.

A notable secondary finding was the differential relationship between systemic inflammation and local myeloid organization. Higher preoperative NLR was associated with an increased CD163/CD68 macrophage-polarization index specifically within the TC, with a significant compartment-by-NLR interaction supporting a spatially restricted association. Although elevated NLR is associated with adverse outcomes in HCC and other malignancies, its biological significance remains uncertain because it reflects multiple components of systemic inflammation (18, 39). Importantly, Lombardi et al. investigated a biologically and clinically distinct population comprising patients with unresectable or advanced HCC receiving first-line systemic treatment with atezolizumab plus bevacizumab, rather than patients undergoing curative-intent resection or transplantation (40). In that systemic-treatment setting, primary refractoriness was associated with elevated NLR and a coordinated immunosuppressive TME characterized by increased myeloid infiltration, enrichment of CD163^+^ TAMs and vascular cancer-associated fibroblasts, a higher Treg/Teff ratio, reduced IFN-γ-related immunogenicity, and pro-tumor cellular interactions. These findings identify the myeloid component of the TME as a major determinant of primary resistance to atezolizumab plus bevacizumab. In our predominantly early-stage surgical cohort, however, neither total NE^+^ density, estimated NE^+^CD177⁻ density, nor the CD177^+^ fraction was associated with NLR. The two studies therefore address different disease stages and therapeutic contexts, and direct extrapolation of the advanced-HCC resistance phenotype to surgically treated HCC is not justified. These findings suggest that peripheral NLR may reflect broader intratumoral myeloid organization rather than the abundance or CD177-defined distribution of individual neutrophil populations, consistent with studies linking systemic inflammation to tumor-associated macrophages and immunosuppressive myeloid states (41, 42). Nevertheless, the association between NLR and intratumoral macrophage composition observed here raises the hypothesis that related myeloid regulatory processes may already operate in earlier-stage disease, but this possibility remains unproven and requires prospective validation.

From a translational perspective, future adjuvant studies in early-stage HCC may benefit from biologically enriched patient selection rather than the unselected application of systemic treatment. Such studies could focus on patients who exhibit both a high clinicopathological risk of recurrence after curative-intent treatment and a TME profile plausibly associated with sensitivity or resistance to immune-checkpoint inhibition. Candidate biomarkers derived from studies of advanced HCC, including IFN-γ-related immune activity and the abundance and spatial organization of CD163^+^ TAMs, vascular cancer-associated fibroblasts, regulatory and effector T cells, and other pro-tumor cellular interactions, could be evaluated prospectively in the adjuvant setting (40). However, these biomarkers have not been validated for treatment selection after curative-intent therapy, and their clinical utility in early-stage surgically treated HCC remains unknown. Moreover, findings from immunotherapy studies in non-transplant populations should not be extrapolated directly to liver-transplant recipients, in whom immune-checkpoint inhibition raises distinct safety concerns.

The tissue findings also provide a spatial context for our previous observation that a higher circulating CD177^+^ neutrophil fraction was independently associated with improved recurrence-free and overall survival following curative treatment for HCC (18). Our previous study evaluated circulating inflammatory-cell indices, whereas the present study examined tissue-based spatial immune indices (18). Postoperative recurrence and survival were not evaluated in the present analysis and therefore, no direct association between the tissue findings and survival outcomes can be inferred. The current finding of a markedly lower CD177^+^ fraction among tumor-associated neutrophils highlights differences in the distribution of CD177-defined populations between circulating and tissue compartments. However, the present data do not establish a direct lineage relationship between these compartments; rather, they suggest that neutrophils encountered within HCC differ markedly in composition from those present in adjacent liver and peripheral blood. This interpretation is consistent with contemporary models in which circulating neutrophils undergo selective recruitment and tissue-specific conditioning, while distinct neutrophil states may also differ in trafficking and survival (8, 9, 31). Moreover, spatial neutrophil remodeling was largely independent of tumor size, grade, multiplicity, BCLC stage, treatment modality, and systemic inflammatory indices after correction for multiple testing. Although the cohort size limits definitive subgroup inference, the consistency of the phenotype across patients suggests that CD177-defined remodeling is not simply a surrogate for tumor burden or conventional disease stage and may instead represent a recurring feature of the local HCC immune environment.

From a translational perspective, the distinct spatial organization of CD177-defined neutrophil populations may merit further investigation. The enrichment of neutrophils within tumor-associated compartments, together with the lower CD177^+^ fraction, indicates that neutrophil populations should not necessarily be considered spatially homogeneous. However, because the present study did not assess cellular function, these findings cannot determine whether the identified populations exert beneficial or detrimental effects in HCC. Functional and mechanistic studies will be required before therapeutic implications can be established. Moreover, the observed association between systemic NLR and the intratumoral CD163/CD68 macrophage-polarization index, but not neutrophil distribution, suggests that circulating inflammatory indices may reflect different components of local myeloid organization and should not be considered interchangeable surrogates of tissue immune composition.

Several strengths and limitations should be considered. The paired spatial design enabled direct within-patient comparison of three biologically distinct compartments while minimizing inter-individual variability. Importantly, the combination of conventional immunohistochemistry and double immunofluorescence on routinely processed FFPE tissue provides a practical and reproducible approach to spatial immune profiling that is potentially applicable beyond centers with access to high-dimensional spatial-omics platforms. However, the retrospective multicenter design and relatively small cohort limit statistical power, particularly for exploratory subgroup analyses. More importantly, the cross-sectional approach cannot determine the biological processes underlying these compartmental differences, and therefore cannot distinguish phenotypic conversion from preferential recruitment, retention, or survival of CD177-defined subsets. A further limitation is that intrasinusoidal/intravascular and extravascular interstitial/intratumoral neutrophils were not classified or quantified separately. Reliable retrospective reclassification cannot be performed using the available immunofluorescence images and corresponding serial H&E sections. Differences in the relative contribution of these micro-anatomical populations among NNL, IM, and TC may therefore have influenced the observed CD177^+^ fractions. Accordingly, the findings apply to the combined NE^+^ population within the analyzed fields and should be interpreted cautiously. CD177 also represents only one dimension of TAN heterogeneity, and estimated NE^+^CD177^−^ density does not provide direct functional characterization of this population, because this subtraction-derived measure depends on both total NE^+^ and CD177^+^NE^+^ quantification, measurement, or classification error in either component may propagate into the estimated value.

Similarly, because CD163 and CD68 were assessed independently on separate tissue sections, the CD163/CD68 macrophage-polarization index represents a relative tissue-level abundance ratio rather than cellular co-expression and cannot establish the polarization state of individual macrophages. CD177 represents only one dimension of TAN heterogeneity. Because the study was based on morphological and marker-based cell quantification without functional assays, it cannot determine whether CD177^+^NE^+^ or estimated NE^+^CD177⁻ neutrophils exert pro-tumor, anti-tumor, or other context-dependent functions. The same limitation applies to the CD68- and CD163-defined macrophage populations. The representative fields used for immunohistochemical quantification were selected independently of the NE-density-driven hotspots used for immunofluorescence. Accordingly, comparisons between neutrophil, macrophage, and vascular measurements reflect compartment-level associations rather than measurements obtained from identical microscopic fields, and direct cross-marker spatial correspondence between the two staining modalities cannot be assumed. Inter-observer agreement was good across the complete evaluated dataset; however, intra-observer agreement was not assessed because repeated evaluations by the same observer were not performed. In addition, the archived FFPE specimens were collected between 2013 and 2024, and block age was not included as a covariate. Time-dependent variation in antigen preservation, particularly for the GPI-anchored surface protein CD177, may therefore have contributed to variability in staining intensity and quantitative detection.

Clinical outcome associations were not evaluated in the present study. Given the limited cohort size and the number of tissue immune indices examined, exploratory recurrence or survival analyses would have been substantially underpowered and vulnerable to unstable estimates and multiple-testing effects. Accordingly, the prognostic value of the spatial neutrophil and macrophage indices remains unknown and should be investigated in a larger cohort with adequate longitudinal follow-up and sufficient outcome events. In addition, the exploratory association between preoperative NLR and the intratumoral CD163/CD68 macrophage-polarization index was not adjusted for potential confounders, including underlying liver disease etiology, fibrosis, or cirrhosis severity, intercurrent infection, and glucocorticoid exposure. These variables may influence both peripheral leukocyte counts and local immune-cell infiltration. Residual confounding therefore cannot be excluded, and the observed association should not be interpreted as causal.

Histological fibrosis stage was not recorded consistently in the retrospective databases and could not be included in the analysis. Accordingly, the NNL compartment denotes liver tissue without HCC and should not be interpreted as histologically normal or fibrosis-free liver. Formal transplant-listing dates were also not uniformly available; therefore, the interval from HCC diagnosis to transplantation was reported as a clinically relevant proxy and should not be interpreted as the formal listing-to-transplant waiting time. Post-transplant immunosuppression was not evaluated because tissue sampling occurred at transplantation, before initiation of post-transplant immunosuppressive treatment, and postoperative outcomes were outside the scope of the present analysis. Prior locoregional treatment and previous hepatic resection were documented, but the cohort size did not permit reliable treatment-stratified or multivariable analyses of their potential effects on tissue immune-cell distributions. The small cohort also precluded reliable multivariable adjustment for multiple clinical covariates without a substantial risk of model overfitting. Future studies combining longitudinal peripheral and tissue sampling with multiplex spatial imaging, single-cell or spatial transcriptomics, and functional characterization of neutrophil and macrophage populations will be required to determine the molecular identity and functional consequences of the spatial populations identified here.

In conclusion, this study indicated that HCC is characterized by marked spatial heterogeneity of the tumor-associated neutrophil compartment. Tumor-associated tissues exhibited substantial neutrophil enrichment accompanied by a pronounced reduction in the CD177^+^ fraction and expansion of estimated CD177^−^ neutrophils, showing distinct compartmental patterns in the relative abundance of CD177-defined neutrophil populations across the HCC microenvironment. Systemic inflammation, reflected by preoperative NLR, was associated with intratumoral CD163/CD68 macrophage polarization but not with neutrophil abundance or CD177-defined distribution, suggesting distinct relationships between systemic inflammation and local myeloid populations. Importantly, these spatial immune patterns were identified using a practical and reproducible tissue-based approach applicable to routinely processed specimens. Beyond their biological relevance, the observed spatial myeloid patterns may provide a framework for more selective immune-modulatory strategies and for a more biologically informed interpretation of systemic inflammatory biomarkers in HCC. Overall, our findings highlight spatial myeloid heterogeneity as a fundamental feature of immune organization in HCC and support further mechanistic investigation of CD177-defined neutrophil populations and their potential therapeutic relevance.

## Data Availability

The raw data supporting the conclusions of this article will be made available by the authors, without undue reservation.

## Supplementary material

Supplementary Figure 1 Distribution of CD8^+^ T cells across hepatocellular carcinoma tissue compartments. CD8^+^ T-cell density (cells/mm²) was quantified in the tumor center (TC), tumor margin/interface (IM), and adjacent non-neoplastic liver (NNL). Each point represents one patient, with gray connecting lines indicating paired measurements across tissue compartments. Boxplots display the median and interquartile range (IQR), with whiskers representing 1.5 × IQR. Overall compartmental differences were assessed using the Friedman test, with Kendall’s coefficient of concordance (Kendall’s W) reported as the effect size. The global mixed-effects model *P* value is shown above the plot.

Supplementary Figure 2 Spatial distribution of CD34^+^ microvascular density across tissue compartments. CD34^+^ microvascular density (vessels/mm²) was quantified in the tumor center (TC), tumor margin/interface (IM), and adjacent non-neoplastic liver (NNL). Each point represents one patient, with gray connecting lines indicating paired measurements across tissue compartments. Boxplots display the median and interquartile range (IQR), with whiskers representing 1.5 × IQR. Overall compartmental differences were assessed using the Friedman test, with Kendall’s W reported as the effect size. The global mixed-effects model *P* value is shown above the plot.

Supplementary Figure 3 Spatial distribution of macrophage populations across hepatocellular carcinoma tissue compartments. Compartment-specific densities (cells/mm²) of (A) CD68^+^ macrophages and (B) CD163^+^ macrophages are shown in the tumor center (TC), tumor margin/interface (IM), and adjacent non-neoplastic liver (NNL). Each point represents one patient, and gray lines connect paired compartmental measurements. Boxplots display the median and interquartile range (IQR), with whiskers representing 1.5 × IQR. Overall compartmental differences were assessed using the Friedman test, with Kendall’s coefficient of concordance (Kendall’s W) reported as the effect size. Global mixed-effects model *P* values are indicated above each panel.

Supplementary Figure 4 Compartment-specific associations between preoperative peripheral-blood neutrophil-to-lymphocyte ratio (NLR) and the IHC-derived CD163/CD68 macrophage-polarization index. Each point represents one patient. Lines are linear visual guides only; statistical inference is based on Spearman rank-correlation analyses.

Supplementary Figure 5 CD163/CD68 macrophage-polarization index according to the predefined preoperative NLR threshold of 3. Each point represents one patient; boxplots display the median and interquartile range (IQR). A nominally higher tumor-center macrophage-polarization index was observed in patients with NLR ≥ 3, whereas no corresponding differences were detected at the tumor margin/interface or in adjacent non-neoplastic liver.

Supplementary Figure 6 Within-patient tumor-center-minus-interface contrast in log_2_(CD163/CD68) according to continuous preoperative NLR. Positive values indicate greater relative enrichment of the CD163/CD68 macrophage-polarization index in the tumor center than in the tumor margin/interface.

## References

[1] Bray F Laversanne M Sung H Ferlay J Siegel RL Soerjomataram I . Global cancer statistics 2022: Globocan estimates of incidence and mortality worldwide for 36 cancers in 185 countries. CA Cancer J Clin. (2024) 74:229–63. doi: 10.3322/caac.21834 38572751

[2] Sung H Ferlay J Siegel RL Laversanne M Soerjomataram I Jemal A . Global cancer statistics 2020: Globocan estimates of incidence and mortality worldwide for 36 cancers in 185 countries. CA Cancer J Clin. (2021) 71:209–49. doi: 10.3322/caac.21660 33538338

[3] Ringelhan M Pfister D O'Connor T Pikarsky E Heikenwalder M . The immunology of hepatocellular carcinoma. Nat Immunol. (2018) 19:222–32. doi: 10.1038/s41590-018-0044-z 29379119

[4] Li J Liang YB Wang QB Luo WL Chen XM Lakang Y . Rechallenge with immune checkpoint inhibitors in patients with hepatocellular carcinoma: A narrative review. Liver Cancer. (2025) 15:611–630. doi: 10.1159/000549355 41409409 PMC12707946

[5] Donne R Lujambio A . The liver cancer immune microenvironment: Therapeutic implications for hepatocellular carcinoma. Hepatology. (2023) 77:1773–96. doi: 10.1002/hep.32740 35989535 PMC9941399

[6] Zhang Q He Y Luo N Patel SJ Han Y Gao R . Landscape and dynamics of single immune cells in hepatocellular carcinoma. Cell. (2019) 179:e20.829–45. doi: 10.1016/j.cell.2019.10.003 31675496

[7] Nemeth T Sperandio M Mocsai A . Neutrophils as emerging therapeutic targets. Nat Rev Drug Discov. (2020) 19:253–75. doi: 10.1038/s41573-019-0054-z 31969717

[8] Arvanitakis K Mitroulis I Germanidis G . Tumor-associated neutrophils in hepatocellular carcinoma pathogenesis, prognosis, and therapy. Cancers (Basel). (2021) 13:1–17. doi: 10.3390/cancers13122899 34200529 PMC8228651

[9] Arvanitakis K Mitroulis I Chatzigeorgiou A Elefsiniotis I Germanidis G . The liver cancer immune microenvironment: Emerging concepts for myeloid cell profiling with diagnostic and therapeutic implications. Cancers. (2023) 15:1522. doi: 10.3390/cancers15051522 36900313 PMC10000678

[10] Grieshaber-Bouyer R Nigrovic PA . Neutrophil heterogeneity as therapeutic opportunity in immune-mediated disease. Front Immunol. (2019) 10:346. doi: 10.3389/fimmu.2019.00346 30886615 PMC6409342

[11] Wang YF Yuan SX Jiang H Li ZX Yin HZ Tan J . Spatial maps of hepatocellular carcinoma transcriptomes reveal spatial expression patterns in tumor immune microenvironment. Theranostics. (2022) 12:4163–80. doi: 10.7150/thno.71873 35673582 PMC9169356

[12] Massoudi D Srivastava A Dhanasekaran R . Unraveling the tapestry: Lessons from multi-omics and spatial biology in hepatocellular cancer. Hepatology. (2026) 84:1229–1245. doi: 10.1097/HEP.0000000000001716 41678777 PMC13629517

[13] Adapa SR Porshe S Talada DP Nywening TM Anderson ML Shaw TI . Spatial transcriptomics reveals distinct architectures but shared vulnerabilities in primary and metastatic liver tumors. Cancers (Basel). (2025) 17:1–20. doi: 10.3390/cancers17193210 41097737 PMC12523610

[14] Li JR Pan X Lin Y Zhao Y Liu Y Li Y . Spatial proximity of immune cell pairs to cancer cells in the tumor microenvironment as biomarkers for patient stratification. Cancers (Basel). (2025) 17:1–16. doi: 10.3390/cancers17142335 40723219 PMC12293128

[15] Van Acker N Frenois FX Gravelle P Tosolini M Syrykh C Laurent C . Spatial mapping of innate lymphoid cells in human lymphoid tissues and lymphoma at single-cell resolution. Nat Commun. (2025) 16:4545. doi: 10.1038/s41467-025-59811-1 40374674 PMC12081901

[16] Zhang M Huang K Yin Q Wu X Zhu M Li M . Spatial heterogeneity of the hepatocellular carcinoma microenvironment determines the efficacy of immunotherapy. Discov Oncol. (2025) 16:15. doi: 10.1007/s12672-025-01747-5 39775241 PMC11706828

[17] Yang D Hu X . Unveiling Cd177: A key player in tumors, autoimmune diseases, and inflammatory disorders. Front Immunol. (2025) 16:1653587. doi: 10.3389/fimmu.2025.1653587 41142798 PMC12546173

[18] Arvanitakis K Pantzios S Christou C Papadopoulos V Theocharidou E Topalidis C . Prognostic significance of neutrophil-to-lymphocyte and platelet-to-lymphocyte ratios in hepatocellular carcinoma: A retrospective multicenter study on overall and recurrence-free survival following liver resection or liver transplantation. Front Immunol. (2026) 17:1801659. doi: 10.3389/fimmu.2026.1801659 42131343 PMC13161088

[19] Arvanitakis K Koletsa T Mitroulis I Germanidis G . Tumor-associated macrophages in hepatocellular carcinoma pathogenesis, prognosis and therapy. Cancers (Basel). (2022) 14:1–22. doi: 10.3390/cancers14010226 35008390 PMC8749970

[20] Arvanitakis K Germanidis G . Immunity revealed: Stratifying hepatocellular carcinoma for precision immunotherapy. Mol Ther Oncol. (2025) 33:200939. doi: 10.1016/j.omton.2025.200939 39990594 PMC11846917

[21] Johnson PJ Dhanaraj S Berhane S Bonnett L Ma YT . The prognostic and diagnostic significance of the neutrophil-to-lymphocyte ratio in hepatocellular carcinoma: A prospective controlled study. Br J Cancer. (2021) 125:714–6. doi: 10.1038/s41416-021-01445-3 34127809 PMC8405698

[22] Lin S Hu S Ran Y Wu F . Neutrophil-to-lymphocyte ratio predicts prognosis of patients with hepatocellular carcinoma: A systematic review and meta-analysis. Transl Cancer Res. (2021) 10:1667–78. doi: 10.21037/tcr-20-3237 35116492 PMC8799187

[23] American College of Radiology Committee on Li-Rads® . Li-Rads Ultrasound Surveillance V2024 Core. Available online at: https://edge.sitecorecloud.io/americancoldf5f-acrorgf92a-productioncb02-3650/media/ACR/Files/RADS/LI-RADS/LI-RADS-US-Surveillance-v2024-Core.pdf (Accessed August 3, 2026).

[24] Reig M Sanduzzi-Zamparelli M Forner A Rimola J Ferrer-Fabrega J Burrel M . Bclc strategy for prognosis prediction and treatment recommendations: The 2025 update. J Hepatol. (2025) 84:631–654. doi: 10.1016/j.jhep.2025.10.020 41151697

[25] Cuschieri S . The strobe guidelines. Saudi J Anaesth. (2019) 13:S31–4. doi: 10.4103/sja.SJA_543_18 30930717 PMC6398292

[26] World Medical A . World Medical Association Declaration of Helsinki: Ethical principles for medical research involving human subjects. JAMA. (2013) 310:2191–4. doi: 10.1001/jama.2013.281053 24141714

[27] Bankhead P Loughrey MB Fernandez JA Dombrowski Y McArt DG Dunne PD . Qupath: Open source software for digital pathology image analysis. Sci Rep. (2017) 7:16878. doi: 10.1038/s41598-017-17204-5 29203879 PMC5715110

[28] Minici R Siciliano MA Ammendola M Santoro RC Barbieri V Ranieri G . Prognostic role of neutrophil-to-lymphocyte ratio (Nlr), lymphocyte-to-monocyte ratio (Lmr), platelet-to-lymphocyte ratio (Plr) and lymphocyte-to-c reactive protein ratio (Lcr) in patients with hepatocellular carcinoma (Hcc) undergoing chemoembolizations (Tace) of the liver: The unexplored corner linking tumor microenvironment, biomarkers and interventional radiology. Cancers (Basel). (2022) 15:1–16. doi: 10.3390/cancers15010257 36612251 PMC9818978

[29] Peng J Chen H Chen Z Tan J Wu F Li X . Prognostic value of neutrophil-to-lymphocyte ratio in patients with hepatocellular carcinoma receiving curative therapies: A systematic review and meta-analysis. BMC Cancer. (2025) 25:571. doi: 10.1186/s12885-025-13972-w 40158082 PMC11954291

[30] Chu HH Kim JH Shim JH Gwon DI Ko HK Shin JH . Neutrophil-to-lymphocyte ratio as a biomarker predicting overall survival after chemoembolization for intermediate-stage hepatocellular carcinoma. Cancers (Basel). (2021) 13:1–11. doi: 10.3390/cancers13112830 34204125 PMC8201147

[31] Arelaki S Koletsa T Sinakos E Papadopoulos V Arvanitakis K Skendros P . Neutrophil extracellular traps enriched with Il-1beta and Il-17a participate in the hepatic inflammatory process of patients with non-alcoholic steatohepatitis. Virchows Arch. (2022) 481:455–65. doi: 10.1007/s00428-022-03330-7 35503185

[32] Ramon-Gil E Geh D Leslie J . Harnessing neutrophil plasticity for Hcc immunotherapy. Essays Biochem. (2023) 67:941–55. doi: 10.1042/EBC20220245 37534829 PMC10539947

[33] Xue R Zhang Q Cao Q Kong R Xiang X Liu H . Liver tumour immune microenvironment subtypes and neutrophil heterogeneity. Nature. (2022) 612:141–7. doi: 10.1038/s41586-022-05400-x 36352227

[34] Jung M Beer A Ghosh S Pylaeva E Alshaar B Tertel T . Cd177 deficiency defines a stable subtype of human neutrophil granulocytes with tumor promoting activity. Adv Sci (Weinh). (2026) 13:e76236. doi: 10.1002/advs.76236 42325214 PMC13336833

[35] Zhou G Peng K Song Y Yang W Shu W Yu T . Cd177+ neutrophils suppress epithelial cell tumourigenesis in colitis-associated cancer and predict good prognosis in colorectal cancer. Carcinogenesis. (2018) 39:272–82. doi: 10.1093/carcin/bgx142 29228136

[36] Yan J Huang A Zhang S Yao N Huang J Jiang Z . Cd177(+) neutrophils drive extracellular matrix remodelling and Hgf-alpha release in Alpps-induced liver regeneration. Gut. (2026) 75:1771–85. doi: 10.1136/gutjnl-2025-336300 41390175 PMC13479642

[37] Wang PC Hu ZQ Zhou SL Yu SY Mao L Su S . The spatial distribution of immune cell subpopulations in hepatocellular carcinoma. Cancer Sci. (2022) 113:423–31. doi: 10.1111/cas.15202 34779545 PMC8819352

[38] Liu Y Xun Z Ma K Liang S Li X Zhou S . Identification of a tumour immune barrier in the Hcc microenvironment that determines the efficacy of immunotherapy. J Hepatol. (2023) 78:770–82. doi: 10.1016/j.jhep.2023.01.011 36708811

[39] Xu C Wu F Du L Dong Y Lin S . Significant association between high neutrophil-lymphocyte ratio and poor prognosis in patients with hepatocellular carcinoma: A systematic review and meta-analysis. Front Immunol. (2023) 14:1211399. doi: 10.3389/fimmu.2023.1211399 37809083 PMC10551132

[40] Lombardi P Ramon-Gil E Raja RQ Brunetti L Manfredi GF Zhou Z . A myeloid immunosuppressive phenotype defines primary refractoriness to atezolizumab plus bevacizumab in hepatocellular carcinoma. J Hepatol. (2026). doi: 10.1016/j.jhep.2026.05.018 42297215

[41] Demyashkin G Atiakshin D Silakov K Shchekin V Bobrov M Vadyukhin M . Spatial distribution and phenotypic profiling of Cd68(+) and Cd163(+) macrophages in melanoma progression: Insights into tumor microenvironment dynamics. Biomedicines. (2025) 13:1–15. doi: 10.3390/biomedicines13092178 41007741 PMC12467158

[42] Kostner AH Nielsen PS Georgsen JB Parner ET Nielsen MB Kersten C . Systemic inflammation associates with a myeloid inflamed tumor microenvironment in primary resected colon cancer-may cold tumors simply be too hot? Front Immunol. (2021) 12:716342. doi: 10.3389/fimmu.2021.716342 34531864 PMC8438238

