# Supplementary material for: Spatial organization of neutrophil and macrophage populations in surgically treated hepatocellular carcinoma: a practical tissue-based immunophenotyping study
Source: Front Immunol. 2026 Oct 7;17:1982507. doi: 10.3389/fimmu.2026.1982507 (PMC13683775; doi:10.3389/fimmu.2026.1982507)
Supplement: Supplementary file 1 [file Table1.docx]

## **Supplementary Tables**

**Table S1.** Primary antibodies, antigen-retrieval conditions, incubation parameters, and detection systems used for immunohistochemistry and dual immunofluorescence

| **Method** | **Target** | **Host and type** | **Clone** | **Manufacturer and catalogue number** | **RRID** | **Working dilution** | **Antigen retrieval** | **Primary-antibody incubation** | **Detection system** |
| --- | --- | --- | --- | --- | --- | --- | --- | --- | --- |
| IHC | CD163 | Mouse monoclonal IgG1 kappa | GHI/61 | Santa Cruz Biotechnology; sc-20066 | Not listed | 1:100 | PT Link; EnVision FLEX High pH, Tris/EDTA, pH 9.0 (K8004); 97°C, 20 min | 20 min at room temperature | EnVision FLEX HRP system (K8023); DAB chromogen; Mayer's hematoxylin counterstain |
| IHC | CD68 | Mouse monoclonal | KP1 | Agilent/Dako; M0814 | AB_2314148 | 1:1,000 | PT Link; EnVision FLEX High pH, Tris/EDTA, pH 9.0 (K8004); 97°C, 20 min | 20 min at room temperature | EnVision FLEX HRP system (K8023); DAB chromogen; Mayer's hematoxylin counterstain |
| IHC | CD8 | Mouse monoclonal | C8/144B | Agilent/Dako; M7103 | AB_2075537 | 1:100 | PT Link; EnVision FLEX High pH, Tris/EDTA, pH 9.0 (K8004); 97°C, 20 min | 20 min at room temperature | EnVision FLEX HRP system (K8023); DAB chromogen; Mayer's hematoxylin counterstain |
| IHC | CD34 | Mouse monoclonal | QBEnd 10 | Agilent/Dako; M7165 | AB_2063006 | 1:100 | PT Link; EnVision FLEX High pH, Tris/EDTA, pH 9.0 (K8004); 97°C, 20 min | 20 min at room temperature | EnVision FLEX HRP system (K8023); DAB chromogen; Mayer's hematoxylin counterstain |
| IF | NE | Rabbit polyclonal | Polyclonal | Atlas Antibodies; HPA066836 | AB_2685733 | 1:200 | Dako Omnis; EnVision FLEX High pH, Tris/EDTA, pH 9.0 (GV804); 97 ± 2°C, 30 min | Overnight at 4°C | Drop-n-Stain CF594 goat anti-rabbit IgG (H+L), highly cross-adsorbed (Biotium, 20955; ready-to-use); 1 h at room temperature, protected from light |
| IF | CD177 | Mouse monoclonal IgG1 kappa | C-5 | Santa Cruz Biotechnology; sc-376329 | AB_10988032 | 1:100 | Dako Omnis; EnVision FLEX High pH, Tris/EDTA, pH 9.0 (GV804); 97 ± 2°C, 30 min | Overnight at 4°C | Mouse IgG Fc-binding protein-CruzFluor 488 (Santa Cruz Biotechnology, sc-533653; 1:200); 1 h at room temperature, protected from light |

*DAB, 3,3'-diaminobenzidine; HRP, horseradish peroxidase; IF, immunofluorescence; IHC, immunohistochemistry; NE, neutrophil elastase; RRID, Research Resource Identifier; RT, room temperature.*

**Table S2.** Exploratory CD163/CD68 macrophage-polarization analysis

| **Parameter** | **n paired patients** | **Tumor center** | **Tumor margin/interface** | **Median paired difference** | **Paired Wilcoxon *P*** |
| --- | --- | --- | --- | --- | --- |
| log₂(CD163/CD68) | 28 | -1.63 (-2.70- -0.95) | -2.71 (-3.69- -1.76) | +0.94 log₂ units | *P* = 0.009 |
| CD163/CD68 index (×100%) | 28 | 32.39 (15.43-51.67) | 15.29 (7.73-29.66) | +11.28 index units | *P* = 0.009 |

*Values are patient-level paired summaries. The CD163/CD68 index should be interpreted as an IHC-derived macrophage-polarization index rather than a direct cellular fraction, because CD68 and CD163 were assessed on separate stains.*

## **Table S3.** Exploratory same-compartment correlations between TAN-related markers and immune/angiogenic markers.

| **Neutrophil-related marker** | **Tissue compartment** | **CD8⁺** | **CD68⁺** | **CD163⁺** | **CD34⁺** |
| --- | --- | --- | --- | --- | --- |
| NE⁺ | Tumor center | 0.15 (0.447) | 0.34 (0.079) | 0.42* (0.027) | -0.44* (0.020) |
| NE⁺ | Tumor margin/interface | -0.24 (0.226) | 0.52* (0.004) | -0.03 (0.878) | -0.40* (0.034) |
| NE⁺ | Non-neoplastic liver | 0.02 (0.911) | -0.20 (0.318) | -0.06 (0.764) | 0.14 (0.463) |
| CD177⁺NE⁺ | Tumor center | 0.01 (0.950) | 0.06 (0.773) | 0.13 (0.522) | -0.03 (0.888) |
| CD177⁺NE⁺ | Tumor margin/interface | -0.08 (0.688) | 0.22 (0.264) | 0.13 (0.508) | -0.25 (0.199) |
| CD177⁺NE⁺ | Non-neoplastic liver | 0.10 (0.602) | 0.00 (0.999) | -0.27 (0.169) | 0.03 (0.872) |
| CD177⁺ fraction among NE⁺ neutrophils | Tumor center | -0.21 (0.287) | -0.09 (0.648) | -0.13 (0.521) | 0.13 (0.497) |
| CD177⁺ fraction among NE⁺ neutrophils | Tumor margin/interface | -0.03 (0.895) | 0.09 (0.632) | 0.19 (0.324) | -0.12 (0.533) |
| CD177⁺ fraction among NE⁺ neutrophils | Non-neoplastic liver | -0.06 (0.761) | -0.04 (0.838) | -0.12 (0.545) | -0.25 (0.194) |

Values are Spearman rho with raw P value in parentheses. * Indicates raw P < 0.05. No correlation remained statistically significant after Benjamini-Hochberg FDR correction for multiple testing. These analyses should therefore be interpreted as exploratory and hypothesis-generating rather than confirmatory.

**Table S4.** Nominal exploratory clinicopathological associations of TAN-related markers.

| **Analysis** | **Biomarker / compartment** | **Clinicopathological variable** | **n** | **Effect / contrast** | **Estimate** | **Raw *P*** | **Holm-adjusted *P*** | **Interpretation** |
| --- | --- | --- | --- | --- | --- | --- | --- | --- |
| Spearman | NE⁺ in Non-neoplastic liver | Tumor grade (numeric) | 28 | rho | -0.41 | 0.036 | 1.000 | Nominal negative monotonic association; not significant after Holm correction. |
| Spearman | CD177⁺ fraction among NE⁺ neutrophils in Non-neoplastic liver | Tumor size (cm) | 28 | rho | -0.42 | 0.025 | 1.000 | Nominal negative monotonic association; not significant after Holm correction. |
| Mann-Whitney | NE⁺ in Non-neoplastic liver | Treatment modality | 28 | Liver transplantation vs Liver resection | 3.75 | 0.049 | 1.000 | Nominal between-group difference in medians; not significant after Holm correction. |
| Mann-Whitney | CD177⁺NE⁺ in Non-neoplastic liver | Treatment modality | 28 | Liver transplantation vs Liver resection | 4.90 | 0.039 | 1.000 | Nominal between-group difference in medians; not significant after Holm correction. |

This table reports only clinicopathological associations with raw P < 0.05 from the exploratory screening analysis. Spearman analyses use rho as the effect estimate; Mann–Whitney analyses report the median difference between the listed groups. No association remained significant after Holm correction; these findings should be interpreted cautiously and used mainly for biological discussion or hypothesis generation.

**Table S5.** Compartment-specific associations between preoperative peripheral-blood neutrophil-to-lymphocyte ratio (NLR) and the CD163/CD68 macrophage-polarization index

| **Compartment** | **Continuous NLR: ρ (p; BH q)** | **NLR ≥3, median (IQR), ×100%** | **NLR <3, median (IQR), ×100%** | **Rank-biserial r** | **Group p (BH q)** |
| --- | --- | --- | --- | --- | --- |
| Tumor center | 0.451 (0.016; 0.048) | 49.1 (23.4–82.9) | 24.3 (12.5–42.0) | 0.456 | 0.043 (0.128) |
| Tumor margin/interface | -0.106 (0.591; 0.591) | 11.5 (7.4–26.7) | 20.9 (9.7–36.0) | -0.159 | 0.490 (0.678) |
| Non-neoplastic liver | 0.245 (0.209; 0.313) | 15.5 (7.6–29.9) | 14.6 (7.3–17.0) | 0.097 | 0.678 (0.678) |

*Values are presented as Spearman's correlation coefficient (ρ) with raw P values and Benjamini–Hochberg (BH)-adjusted q values for analyses using continuous preoperative NLR. Group comparisons were performed using the Wilcoxon rank-sum test according to the preoperative NLR threshold of 3, with rank-biserial correlation reported as the corresponding effect-size measure. The CD163/CD68 index should be interpreted as an immunohistochemistry-derived macrophage-polarization index rather than a direct cellular fraction because CD163 and CD68 were assessed on separate tissue sections.*

**Table S6.** Generalized estimating equation analysis demonstrating compartment-specific associations between preoperative peripheral-blood NLR and the CD163/CD68 macrophage-polarization index

| **Model** | **Effect** | **log₂ estimate (95% CI)** | **Fold effect (95% CI)** | **p** |
| --- | --- | --- | --- | --- |
| Continuous NLR (per 1 SD) | Tumor center | 0.654 (0.261–1.047) | 1.57 (1.20–2.07) | 0.001 |
| Continuous NLR (per 1 SD) | Tumor margin/interface | 0.013 (-0.321–0.348) | 1.01 (0.80–1.27) | 0.937 |
| Continuous NLR (per 1 SD) | Non-neoplastic liver | 0.126 (-0.489–0.742) | 1.09 (0.71–1.67) | 0.687 |
| Continuous NLR (per 1 SD) | Tumor center minus interface | 0.641 (0.102–1.180) | 1.56 (1.07–2.27) | 0.020 |
| Continuous NLR (per 1 SD) | Tumor center minus non-neoplastic liver | 0.528 (0.010–1.046) | 1.44 (1.01–2.07) | 0.046 |
| NLR ≥3 vs <3 | Tumor center | 1.199 (0.256–2.141) | 2.30 (1.19–4.41) | 0.013 |
| NLR ≥3 vs <3 | Tumor margin/interface | -0.340 (-1.166–0.486) | 0.79 (0.45–1.40) | 0.420 |
| NLR ≥3 vs <3 | Non-neoplastic liver | 0.379 (-0.616–1.374) | 1.30 (0.65–2.59) | 0.456 |
| NLR ≥3 vs <3 | Tumor center minus interface | 1.539 (0.481–2.597) | 2.91 (1.40–6.05) | 0.004 |
| NLR ≥3 vs <3 | Tumor center minus non-neoplastic liver | 0.820 (-0.359–1.998) | 1.77 (0.78–4.00) | 0.173 |

*Generalized estimating equations (GEE) with exchangeable within-patient correlation were used to evaluate whether the association between preoperative NLR and the CD163/CD68 macrophage-polarization index differed across tissue compartments. Continuous NLR was modelled per one standard deviation (SD) increase. Effect estimates are presented on the log₂ scale together with back-transformed fold changes and corresponding 95% confidence intervals (CI). Interaction rows represent within-patient comparisons between tissue compartments. Overall compartment-by-continuous-NLR interaction: Wald χ² = 10.41 (2 degrees of freedom), P = 0.005. Overall compartment-by-NLR-group interaction: Wald χ² = 8.38 (2 degrees of freedom), P = 0.015.*

**Table S7.** Compartment-specific associations between preoperative peripheral-blood NLR and the CD177^+^ neutrophil fraction

| **Tissue compartment** | ***n*** | **Continuous NLR, Spearman's *ρ*** | **Raw *P* value** | **BH-adjusted *q* value** | **NLR ≥3 Median (IQR), %** | **NLR <3 Median (IQR), %** | **Rank-biserial *r*** | **Raw *P* value** | **BH-adjusted *q* value** |
| --- | --- | --- | --- | --- | --- | --- | --- | --- | --- |
| Tumor center | 28 | 0.033 | 0.866 | 0.866 | 12.25 (0.54–89.38) | 23.19 (3.81–51.48) | −0.010 | 0.982 | 0.982 |
| Tumor margin/interface | 28 | −0.132 | 0.504 | 0.756 | 6.61 (1.18–17.29) | 13.59 (0.00–23.33) | −0.082 | 0.727 | 0.982 |
| Non-neoplastic liver | 28 | 0.312 | 0.106 | 0.318 | 90.00 (68.82–100.00) | 87.59 (73.52–97.79) | 0.103 | 0.654 | 0.982 |

*Values represent the percentage of CD177***^+^** *neutrophils among total NE***^+^** *neutrophils. Continuous associations between preoperative peripheral-blood NLR and the CD177***^+^** *neutrophil fraction were evaluated using Spearman's rank correlation. Group comparisons were performed using the Wilcoxon rank-sum test according to the preoperative NLR threshold of 3, with rank-biserial correlation reported as the corresponding effect-size measure. Benjamini–Hochberg (BH) adjustment was applied separately to the three compartment-specific correlation analyses and the three group comparisons. No statistically significant associations were identified, supporting the absence of a compartment-specific relationship between systemic inflammatory status and the distribution of CD177-defined neutrophil populations in this exploratory cohort.*
